# The difference between LSMC and replicating portfolio in insurance liability modeling

**DOI:** 10.1007/s13385-016-0133-z

**Published:** 2016-11-04

**Authors:** Antoon Pelsser, Janina Schweizer

**Affiliations:** 1 Departments of Quantitative Economics and Finance, Maastricht University, Netspar, Kleynen Consultants, P.O. Box 616, 6200 MD Maastricht, The Netherlands; 2 Department of Quantitative Economics, Maastricht University, Netspar, P.O. Box 616, 6200 MD Maastricht, The Netherlands

**Keywords:** Portfolio replication, Least squares Monte Carlo, Least squares regression

## Abstract

Solvency II requires insurers to calculate the 1-year value at risk of their balance sheet. This involves the valuation of the balance sheet in 1 year’s time. As for insurance liabilities, closed-form solutions to their value are generally not available, insurers turn to estimation procedures. While pure Monte Carlo simulation set-ups are theoretically sound, they are often infeasible in practice. Therefore, approximation methods are exploited. Among these, least squares Monte Carlo (LSMC) and portfolio replication are prominent and widely applied in practice. In this paper, we show that, while both are variants of regression-based Monte Carlo methods, they differ in one significant aspect. While the replicating portfolio approach only contains an approximation error, which converges to zero in the limit, in LSMC a projection error is additionally present, which cannot be eliminated. It is revealed that the replicating portfolio technique enjoys numerous advantages and is therefore an attractive model choice.

## Introduction

**Fig. 1:**
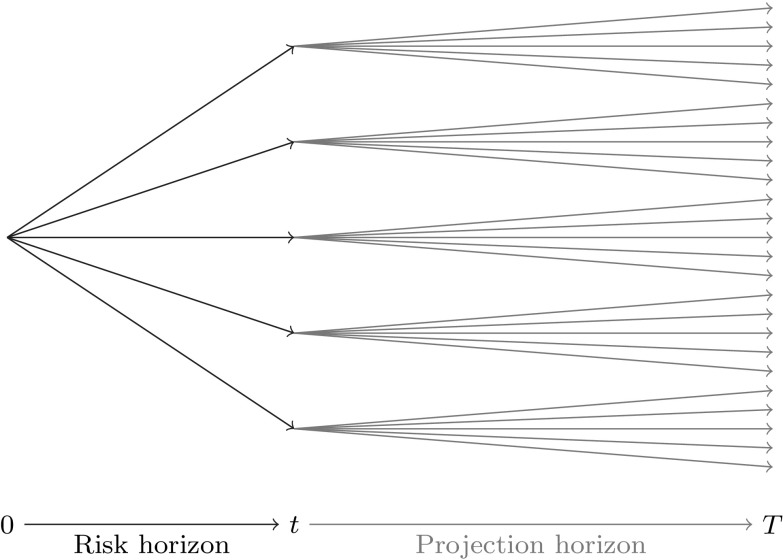
Nested stochastic simulation problem

The Solvency II framework requires insurers to appropriately evaluate and manage embedded balance sheet risks. In the context of calculating risk figures, insurers are challenged to revalue their liabilities under economic stress scenarios based on fair market valuation principles (see Article 76, [40]). Particularly for life insurance liabilities, which contain embedded options and guarantees coming from policyholder participations, minimum guarantees and surrender options, this leaves the insurer with a strenuous task. As a consequence, numerical methods involving Monte Carlo techniques for estimating the value of the liabilities have gained much attention. Procedures known as “nested simulation” or “full stochastic Monte Carlo simulation” take a full simulation approach, from which the empirical distribution of the liability values at the relevant point in time *t* is obtained. In insurance risk reporting, *t* typically corresponds to 1 year. Based on the empirical distribution, the estimate for the *t* year value at risk (VaR) can be derived, which is the Solvency II relevant risk figure. The nested simulation approach is illustrated in Fig. 1, where the first simulation set from time 0 to time *t* represents the real-world scenarios over the risk horizon, and the second set from time *t* to time *T* gives the risk-neutral scenarios for the estimation of the value at time *t*; see also [4, 8].

Due to the scale and scope of a typical insurer’s life liabilities, the nested stochastic simulation approach is computationally inefficient and, regarding relevant reporting on the risk situation of the insurance company, a too timely exercise. For that reason, alternative methods have been explored, which combine approximation methods with Monte Carlo techniques with the ambition to yield accurate risk capital figures within a reasonable time frame. Major discussions among practitioners revolve around two of these methods, largely known as portfolio replication and least squares Monte Carlo (LSMC) (see, e.g., [4, 33, 35]). In this paper, we want to shed light on the differences between these two approaches and the practical consequences that result.

LSMC originates from the idea of estimating the continuation value of an American option through cross-sectional regression on Monte Carlo simulated paths. By going backward in time, the American option price can thus be determined. Examples for LSMC in the context of American option pricing can be found in [5, 12, 15, 19–21, 30, 39, 41, 42]. Andreatta and Corradin [1] and Bacinello et al. [2, 3] apply the LSMC approach to the valuation of life insurance policies with surrender options. Devineau and Chauvigny [18] show how the LSMC method can be extended to obtain a portfolio of replicating assets consisting of standard financial instruments. All these authors have in common that the static representations that are constructed immediately estimate the valuation function rather than the payoff function of the contingent claim. In the context of the insurance problem of estimating the risk capital at time *t*, this means that the LSMC method yields an approximation function for the conditional expectation function at time *t*. This allows the rapid obtaining of an empirical distribution of the time *t* value under different real-world scenarios, from which the risk capital figure can then be extracted.

Glasserman and Yu [21] were the first to offer a different perspective on the LSMC method. They describe LSMC techniques that directly estimate the valuation function “regression now” and propose a slightly different approach termed “regression later”. In “regression later”, the terminal payoff of the contingent claim is first approximated by a linear combination of basis functions. The approximation to the valuation function at time *t* is then attained by evaluating the basis functions under the conditional expectation operator at time *t*. Both LSMC types, regress-now and regress-later, have been further investigated in [7]. Moreover, in [8], it has been shown that the LSMC regress-later approach corresponds to the replicating portfolio technique. The principle of static replication is to construct a portfolio of financial instruments that mirrors the terminal payoff function of a target random variable. The static replicating portfolio is perfect if it replicates the target payoff in every possible state of the world. By the no-arbitrage condition, if the payoff of the target security is perfectly replicated, the replication automatically matches the security’s value at all times prior to maturity, implying that they have the same market-consistent price. Given a replicating portfolio to the payoff of a contingent claim consisting of instruments for which its values are readily available, the time *t* value can be quickly determined under different real-world scenarios, which again allows the extraction of risk capital figures. Naturally, this feature has been exploited in the risk management of life insurance liabilities. Pelsser [36] leverages the static portfolio replication concept to derive hedging strategies with swaptions for life insurance policies with guaranteed annuity options. Oechslin et al. [35] consider how to set up replicating portfolios for life insurance liabilities in a more generalized approach. Recently, Natolski and Werner [33] discuss and compare several approaches to the construction of replicating portfolios in life insurance. Chen and Skoglund [13], Daul and Vidal [17], Kalberer [26], Koursaris [28, 29], and Burmeister [11], for example, address the construction of replicating portfolios in life insurance from a more practical point of view and make recommendations. Taking the replicating portfolio as a proxy to the true liability payoff or the LSMC estimator as a proxy to the liability value at time *t* speeds up risk calculations tremendously. Thus, both methods fulfill the target of enabling risk capital calculations for a life insurance portfolio. The straightforward question is thus which method to use and why. The current literature offers little insight into what are the essential differences between these methods and their advantages over the other. Glasserman and Yu [21] compare the properties of the coefficient estimates given that the approximations attained with LSMC regress-now and with LSMC regress-later yield a linear combination of the same basis functions. Their results suggest that in a single-period problem the LSMC regress-later algorithm yields a higher coefficient of determination and a lower covariance matrix for the estimated coefficients; see also [10] in which similar observations are reported. Beutner et al. [7] remark that the functions to be approximated in LSMC regress-now may differ in nature compared to LSMC regress-later. Examples are provided which underline this observation. Several practitioners have touched on a qualitative assessment of the advantages and disadvantages of particular proxy techniques, including LSMC and portfolio replication; see, for example, [23, 24, 27, 32]. While all these authors contribute to the discussion on the differences between LSMC and portfolio replication, no structured framework is provided to explain the observations. We attempt to close this gap with this paper.

In this paper, we want to give insight into the fundamental differences between LSMC and portfolio replication. As has already been pointed out, the replicating portfolio estimator corresponds to LSMC regress-later. When we use the brief terminology “LSMC”, we refer to the regress-now type. Both are regression-based Monte Carlo methods, but we will accentuate that one is a function-fitting method while the other is truly a portfolio replication approach. As we will see, this allows us to implement a simple measure in portfolio replication as a valuable indicator for the quality of the replicating portfolio. First, the mathematical models for both approaches are presented, based on which are the fundamental differences between the two methods to be pinned down. Then, we will elaborate on the consequences that follow from the difference between these methods. We will illustrate our conclusions with straightforward examples, which are simple but compelling. Finally, we will address the challenges that arise for path-dependent insurance products.

The structure of this paper is as follows. In Sect. 2, we repeat the mathematical framework for LSMC and portfolio replication, which is largely taken from [7]. We will highlight the mathematical difference between these two models, which builds the basis for the sections to follow. In Sect. 3, we elaborate on the consequences that result from the difference between LSMC and portfolio replication. In Sect. 4, the challenges for path-dependent payoff functions are addressed. Section 5 concludes.

## The regression model for LSMC and portfolio replication

In this section, we give the mathematical model and the estimation approach for the LSMC and the portfolio replication techniques. We will see that the approaches are very similar but differ in one significant aspect. Both the model and the notation largely follow [7], which we repeat here.

Life insurance liabilities commonly generate several stochastic payoffs at different time points on a finite time horizon. The stochastic payoffs are typically driven by finitely many underlying risk drivers, which may be of both a financial as well as a nonfinancial nature. For our model, we fix a finite time horizon *T*. We denote the terminal payoff of an insurance contingent claim at time *T* by *X*, which is driven by a *d*-dimensional stochastic process *Z*. We define the terminal cash flow as the sum of all cash flows over time [0, *T*] accumulated in the money market account to the time point *T*. This is in line with the definitions in [31, 35]. Let us now define the underlying dynamics of the contingent payoff *X*. Consider $Z=\{Z(t), 0 \le t \le T\}$ to be a *d*-dimensional stochastic process with $d \in {\mathbb {N}}$ defined on some filtered probability space $(\Omega ,\mathcal {F},\lbrace \mathcal {F}_t \rbrace _{0 \le t \le T}, \tilde{\mathbb {P}})$. We denote the filtration generated by *Z* by $\{\mathcal {F}_t\}_{0 \le t \le T}$. The measure $\tilde{\mathbb {P}}$ denotes some probability measure equivalent to the true probability measure $\mathbb {P}$. We interpret *Z* to be the ultimate *d*-dimensional random driver, on which the cash flows of an insurance contingent claim depend. We do not further specify *Z*, but remark that in principle it may account for both financial and nonfinancial risks. The paths $Z(\cdot ,\omega )$ with $\omega \in \Omega$, of *Z* given by $t \rightarrow Z(t,\omega )$, $t \in [0,T]$, are assumed to lie in some function space $\mathbb {D}_d[0,T]$ consisting of functions mapping from [0, *T*] to $\mathbb {R}^d$, and we consider *Z* as a random function. Recall that the payoff function *X* is driven by *Z*. We assume that the payoff *X* is $\mathcal {F}_T$-measurable and we want to write *X* in terms of *Z*. However, as insurance contingent claims are typically path-dependent and generate multiple cash flows over time, the payoff *X* at time *T* depends on the paths of $Z(\cdot ,\omega )$. Thus, we define a process, denoted by $A_T(Z)$, which carries all the information on the paths of the *d*-dimensional stochastic process *Z* from time 0 to *T* which is relevant for the contingent claim *X*. We denote the dimensionality of $A_T$ by $\ell _T$, which is driven by the dependence structure on the *d*-dimensional process *Z* and the number of characteristics on the stochastic path that are required to determine *X*. Now, we can write for every $\omega$ in the sample space $\Omega$ the payoff $X(\omega )$ of the contingent claim *X* as $g_T(A_T(Z(\cdot ,\omega )))$, where $A_T$ is a known (measurable) functional mapping from the function space $\mathbb {D}_d[0,T]$ to $\mathbb {R}^{\ell _T}$ and $g_T$ is a known Borel-measurable function that maps from $\mathbb {R}^{\ell _T}$ to $\mathbb {R}$. Note that if we were only interested in plain vanilla contingent claims at time *T*, it would suffice to observe the stochastic process *Z* at time *T*, but as insurance liabilities are often path-dependent, we need the information on the process of the underlying risk factors over time that is relevant for the contingent claim *X*, which we store in $A_T(Z)$.

The characterization of $A_T(Z)$ is subject to the specification of the modeler. Take the example of an Asian option with maturity *T*, where *X* gives the payoff of the Asian option at its maturity date *T*. In order to get the payoff, it suffices to observe the time average of the underlying over the run-time of the Asian option. This information would be stored in $A_T(Z)$ and we would have $\ell _T=1$. Alternatively, we may also observe the values of the underlying at each time point, which we would store in $A_T(Z)$. Then, $\ell _T=T$. From this example, we can see that $A_T(Z)$ is not unique but depends on the choice of the modeler. We will return to this topic in Sect. 4.

As in [7], we restrict attention to finite second-moment contingent claims and refer to the relevant related literature, in which the same assumption is applied (see, , [6, 30, 31, 39]). Thus, we assume that the contingent claim *X* has finite mean and variance, which allows us to model it as an element of a Hilbert space (see also [31]). More specifically, we assume that $g_T$ belongs to the functional space $L_2\big (\mathbb {R}^{\ell _T}, \mathcal {B}(\mathbb {R}^{\ell _T}), \tilde{\mathbb {P}}^{A_T(Z)}\big )$, where $\mathcal {B}(\mathbb {R}^{\ell _T})$ denotes the Borel $\sigma$-algebra on $\mathbb {R}^{\ell _T}$, and $\tilde{\mathbb {P}}^{A_T(Z)}$ denotes the probability measure on $\mathbb {R}^{\ell _T}$ induced by the mapping $A_T(Z)$. Now, $L_2(\mathbb {R}^{\ell _T}, \mathcal {B}(\mathbb {R}^{\ell _T}), \tilde{\mathbb {P}}^{A_T(Z)})$ is a separable Hilbert space with inner product

$$\begin{aligned} \int _{\mathbb {R}^{\ell _T}} h_1(u)h_2(u)\, \mathrm {d}\tilde{\mathbb {P}}^{A_T(Z)}(u)=\mathbb {E}_{\tilde{\mathbb {P}}}[h_1(A_T(Z))h_2(A_T(Z))] \end{aligned}$$

and norm

$$\begin{aligned} \sqrt{\int _{\mathbb {R}^{\ell _T}} h_1(u)h_1(u)\, \mathrm {d}\tilde{\mathbb {P}}^{A_T(Z)}(u)}=\sqrt{\mathbb {E}_{\tilde{\mathbb {P}}}[h_1^2(A_T(Z))]} \end{aligned}$$

[9]. Recall that a Hilbert space simply abstracts the finite-dimensional geometric Euclidean space to infinite dimensions [16]. The theory for constructing the LSMC and the portfolio replication estimates is largely driven by the fact that, under the restriction to finite variance contingent claims, the payoff *X* is an element of a separable Hilbert space. This allows us to express it in terms of a countable orthonormal basis. We will elaborate on the details in Sects. 2.1 and 2.2, where the least squares regression models for LSMC and replicating portfolios, respectively, are presented.

Recall our initial problem of calculating risk figures. An insurer that needs to calculate the risk capital for its life insurance portfolio is ultimately interested in obtaining the empirical distribution for the values of *X* at the risk horizon $t \le T$, where *t* typically corresponds to 1 year in the Solvency II framework. Basically, the insurer is interested in the expectation of *X* conditional on information at time *t*. The nested stochastic simulation approach discussed in Sect. 1 is one path to obtain a solution to the problem. However, as previously pointed out, the simulation effort is too high and in that respect the nested simulation approach is infeasible. LSMC and portfolio replication both reduce the simulation effort by requiring a smaller amount of inner simulations in Fig. 1 to obtain an approximating function to the conditional expectation of *X*. However, they differ very much in the way that the approximating function is constructed. While in LSMC an approximating function to $\mathbb {E}_{\tilde{\mathbb {P}}}[X | \mathcal {F}_t]$ is directly yielded through a least squares regression, portfolio replication focuses instead on approximating the payoff function *X*. This approximation is also obtained through least squares regression, but with different regressors than in LSCM. Given the approximating function for *X*, its conditional expectation is estimated by applying the conditional expectation operator to the approximating function. This implies that regressors for the approximation to *X* must be chosen, for which the conditional expectation is either exact or can be quickly and fairly accurately estimated through numerical integration. Taking the above into account, we will explain in the following two sections the least squares approaches for constructing the LSMC and the portfolio replication estimates.

### Least squares Monte Carlo

The least squares Monte Carlo (LSMC) method has received much attention in the academic literature, particularly in the context of estimating the continuation value in American option pricing; see, for example, [30, 41] and also [39]. Calculating risk capital figures for life insurance portfolios poses a similar problem to the extent that an unknown conditional expectation function must be estimated. Therefore, the LSMC method has also found its appeal in insurance risk modeling. Importantly, in LSMC, the estimation of the conditional expectation function is achieved in one step by exploiting the cross-sectional information in Monte Carlo simulations and regressing across time using least squares. To describe the LSMC approach, we assume that the quantity of interest, $\mathbb {E}_{\tilde{\mathbb {P}}}[X|\mathcal {F}_t]$, can be written as

2.1 $$\begin{aligned} g_{0,t}\big (A_t(Z)\big ) = \mathbb {E}_{\tilde{\mathbb {P}}}\left[ X|\mathcal {F}_t\right] , \quad 0\le t < T, \end{aligned}$$

where $A_t$ is a known (measurable) functional mapping from $\mathbb {D}_d[0,t]$ to $\mathbb {R}^{\ell _t}$ and $g_{0,t}$ is an unknown Borel-measurable function that maps from $\mathbb {R}^{\ell _t}$ to $\mathbb {R}$. Here, $\mathbb {D}_d[0,t]$ is the restriction of $\mathbb {D}_d[0,T]$ to the interval [0, *t*] and $\ell _t$ denotes the dimensionality of $A_t(Z)$.

#### Remark 1

We use $g_{0,t}(A_t(Z))$ to denote the expected time *t* value of *X*, which is generally unknown. The subscript “0” is deliberately used to contrast the conditional expectation as an unknown function from the payoff function $g_T(A_T(Z))$, which is known in a simulation-based model as the simulation is controlled by the modeler.

In the following, we describe the LSMC approach for estimating $g_{0,t}$. Recall that the square-integrability of *X* implies that $\mathbb {E}_{\tilde{\mathbb {P}}}[X|\mathcal {F}_t]$ is also square-integrable. Hence, we also have that $g_{0,t} \in L_2\big (\mathbb {R}^{\ell _t}, \mathcal {B}(\mathbb {R}^{\ell _t}), \tilde{\mathbb {P}}^{A_t(Z)}\big )$, which is again a separable Hilbert space. It is a well-known result that a separable Hilbert space has a countable orthonormal basis, in terms of which its elements may be expressed; see, for instance [9], Corollary 4.2.2 and Corollary 4.3.4]. Then, we can write $g_{0,t}$ as

$$\begin{aligned} g_{0,t} = \sum _{k=1}^{\infty } \beta _k v_k, \end{aligned}$$

where $\left\{ v_k \right\} _{k=1}^{\infty }$ is a countable orthonormal basis of the Hilbert space, in which $g_{0,t}$ lies. Because $g_{0,t}$ is the projection of *X*, the coefficients are given as

2.2 $$\begin{aligned} \beta _k = \mathbb {E}_{\tilde{\mathbb {P}}}[\mathbb {E}_{\tilde{\mathbb {P}}}[X | \mathcal {F}_t] v_k(A_t(Z))] = \mathbb {E}_{\tilde{\mathbb {P}}}[X v_k(A_t(Z))]. \end{aligned}$$

Thus, in particular, we have

2.3 $$\begin{aligned} g_{0,t}\left( A_t(Z)\right) = \sum _{k=1}^{\infty } \beta _k v_k \left( A_t(Z)\right) . \end{aligned}$$

and, as usual, we define the projection error $p_{0,t}$ by

2.4 $$\begin{aligned} p_{0,t}(A_T(Z)):=X-g_{0,t}(A_t(Z)). \end{aligned}$$

The LSMC approach tries to estimate the unknown function $g_{0,t}$ through its representation in Eq. (2.3) by generating data under $\tilde{\mathbb {P}}$. However, Eq. (2.3) involves infinitely many parameters, which leaves a direct estimation infeasible. Consequently, finite-dimensional approximations with a truncated basis $\lbrace v_k \rbrace _{k=1}^K$, $K < \infty$, are used instead. For Eq. (2.3) this implies that with sieves we approximate $g_{0,t}$ by

2.5 $$\begin{aligned} g_{0,t}^K := \sum _{k=1}^K \beta _k v_k = \left( \varvec{\beta }_K \right) ^T \varvec{v}_K, \end{aligned}$$

where $\varvec{\beta }_K=( \beta _1,\ldots ,\beta _K )^T$, $\varvec{v}_K=( v_1,\ldots ,v_K)^T$, and *T* denotes transpose. Thus, a superscript ${}^T$ means transpose and it should be easy to distinguish it from the terminal time *T*. This results in an approximation error $a_{0,t}^K$ for $g_{0,t}$ given by

2.6 $$\begin{aligned} a_{0,t}^K:=g_{0,t}-g_{0,t}^K, \end{aligned}$$

Notice that we have $\mathbb {E}_{\tilde{\mathbb {P}}}[ g_{0,t}^K(A_t(Z))a_{0,t}^K(A_t(Z))]=0$ by construction.^1^ By definition, the approximation error $a_{0,t}^K$ converges to zero as $K \rightarrow \infty$. We can now write the following regression equation

2.7 $$\begin{aligned} X= g_{0,t}^K(A_t(Z)) + a_{0,t}^K(A_t(Z))+p_{0,t}(A_T(Z)), \end{aligned}$$

where the sum of the approximation and the projection error represents the regression error. Now, given a (simulated) sample of size *N* denoted by

$\big ((x_1,A_t(z_1)),\ldots ,(x_N,A_t(z_N))\big )$ , it is natural to estimate $g_{0,t}^K$ by the ‘sample projection’

$$\begin{aligned} \hat{g}_{0,t}^K = \arg \min _{g \in \mathcal {H}_K} \frac{1}{N} \sum _{n=1}^N \left( x_n - g(A_t(z_n)) \right) ^2, \end{aligned}$$

where $\mathcal {H}_K:=\left\{ g: \mathbb {R}^{\ell _t} \rightarrow \mathbb {R}\;| \; g=\sum _{k=1}^K \beta _k v_k, \beta _k \in \mathbb {R}\right\}$. This corresponds to the least squares estimation of the above regression equation, i.e. from regressing the time *T* payoff of the contingent claim *X* against *K* explanatory variables valued at time *t*.

Thus, we have

2.8 $$\begin{aligned} \hat{g}_{0,t}^K = \left( \varvec{\hat{\beta }}_K \right) ^T\mathbf {v}_K, \end{aligned}$$

with

$$\begin{aligned} {\hat{\varvec{\beta }}}_K = \left( \left( \varvec{V}_K \right) ^T\varvec{V}_K \right) ^{-1} \left( \varvec{V}_K\right) ^T\varvec{X}, \end{aligned}$$

where $\varvec{X} = \left( x_{1},\ldots , x_{N}\right) ^T$ and $\varvec{V}_K$ is an $N \times K$ matrix with the *n*th row equal to $\varvec{v}_K(A_t(z_{n}))$, $n=1,\ldots ,N$.

**Fig. 2:**
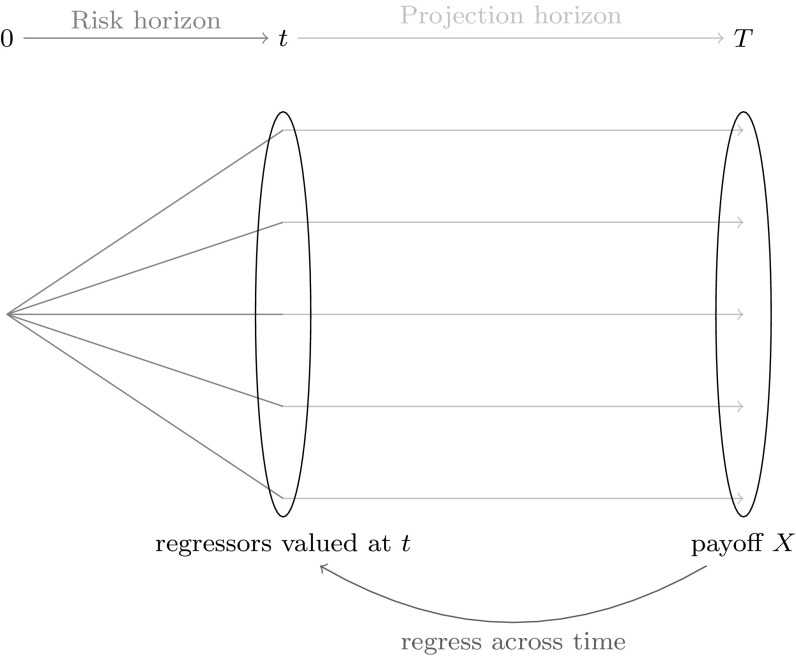
Illustration of the LSMC approach

We illustrate the LSMC approach in Fig. 2. Based on calibration scenarios, the LSMC estimator is constructed by regressing the payoff function *X* against regressors valued at time *t*. The least squares regression approach naturally provides thereby an estimate for the conditional expectation function $\mathbb {E}_{\tilde{\mathbb {P}}}[X | \mathcal {F}_t]$. Given this estimate, the distribution of time *t* values over real-world scenarios constructed on the risk horizon can be obtained.

Naturally, the LSMC estimator is subject to an error. More specifically, the LSMC estimator $\hat{g}_{0,t}^K$ involves three sources of error resulting from an approximation, a projection and an estimation error. This can also be seen nicely from Eq. (2.5), which gives the regression equation. The regression error here consists of the approximation and the projection error. The estimation error arises from estimating the coefficients of the regression equation based on a finite sample. While the approximation error vanishes for $K \rightarrow \infty$ and the estimation error for $N \rightarrow \infty$, the projection error cannot be eliminated in the limit. The nonzero projection error arises from projecting the cash flows across the time interval [*t*, *T*]. To better see the impact of the projection error on the estimation result, consider the coefficient error,

$$\begin{aligned} ( {\hat{\varvec{\beta }}}_K - \varvec{\beta }_K)&= ((\varvec{V}_K )^T\varvec{V}_K )^{-1} (\varvec{V}_K)^T (\varvec{X} - \varvec{V}_K \varvec{\beta }_K) \\&= ((\varvec{V}_K )^T\varvec{V}_K)^{-1} (\varvec{V}_K)^T (( \varvec{X} - \varvec{V} \varvec{\beta } ) + (\varvec{V} \varvec{\beta } - \varvec{V}_K \varvec{\beta }_K ) ) \\&= ((\varvec{V}_K )^T\varvec{V}_K )^{-1} (\varvec{V}_K)^T ( \varvec{p}_{0,t} + \varvec{a}_{0,t}^K) \end{aligned}$$

Observe that the projection error can in fact only be eliminated by regressing the payoff *X* valued at time *T* against regressors valued at the same time point. This brings us to the replicating portfolio approach, which we address in the following section.

### Portfolio replication

In the previous section, we have discussed the LSMC approach, which obtains an estimate to the time *t* value of a contingent claim by regressing the payoffs at time *T* resulting from a Monte Carlo simulation sample against basis functions valued at time *t*. In contrast, in this section, we are first interested in constructing an estimate to the payoff function *X*, i.e. we construct a static replicating portfolio to the payoff function. Then, given the linear representation of *X* through basis functions, apply the operator $\mathbb {E}_{\tilde{\mathbb {P}}}\left[ \cdot |\mathcal {F}_t\right]$ to these basis functions. The approach takes advantage of the linearity of the expectation operator. Note that the two-step approach is advantageous if basis functions are used for the payoff function *X* whose conditional expectation is easily obtained. For the case where $\tilde{\mathbb {P}} = \mathbb {Q}$ with $\mathbb {Q}$ denoting the risk-neutral measure, this implies that closed-form solutions for the price of the basis functions must be readily available. The replicating portfolio approach corresponds to the LSMC regress-later approach first discussed in [21]; see also [7].

Remember that we assume square-integrability of the payoff function, meaning that $g_T \in L_2\big (\mathbb {R}^{\ell _T}, \mathcal {B}(\mathbb {R}^{\ell _T}), \tilde{\mathbb {P}}^{A_T(Z)}\big )$. Hence, by the same argument as in Sect. 2.1,

2.9 $$\begin{aligned} X=g_{T}(A_T(Z)) = \sum _{k=1}^{\infty } \alpha _k e_k(A_T(Z)), \end{aligned}$$

where $\left\{ e_k \right\} _{k=1}^{\infty }$ is a countable orthonormal basis of $L_2\big (\mathbb {R}^{\ell _T}, \mathcal {B}(\mathbb {R}^{\ell _T}), \tilde{\mathbb {P}}^{A_T(Z)}\big )$.

**Fig. 3:**
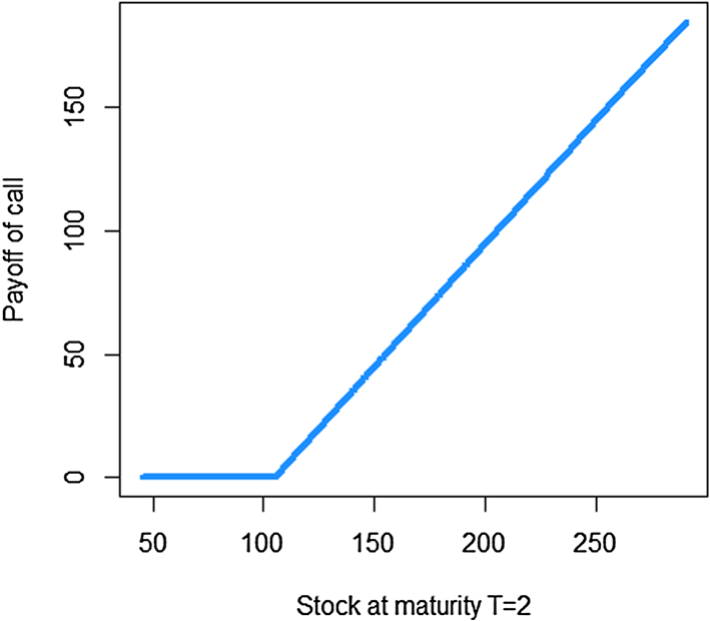
Payoff function for a call with maturity $T=2$

**Fig. 4:**
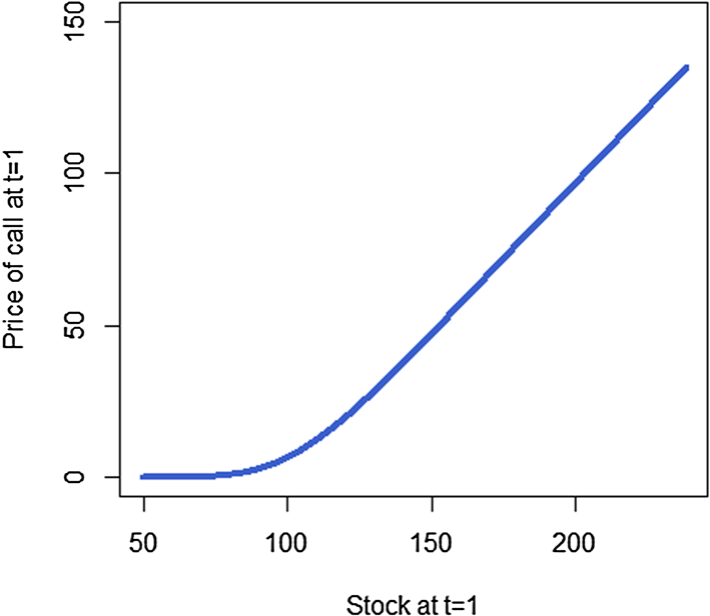
Pricing function at $t=1$ for call with maturity $T=2$

We use a different notation for the coefficients and the basis functions than in Sect. 2.1 to emphasize that, in general, the basis functions chosen for LSMC may differ from the ones used in portfolio replication, the reason being that the functions to be approximated in LSMC and in portfolio replication may differ in nature. Recall that in LSMC we directly estimate the conditional expectation function, while in portfolio replication the approximation refers to the payoff function. Take the example of a call option. The payoff has a kinked structure, but the conditional expectation function is smooth (see Figs. 3, 4). Thus, for that specific example, polynomials are a convenient basis in LSMC to approximate the smooth conditional expectation function, while for the payoff function piecewise linear functions are, for instance, more appropriate in order to replicate the kink.

The coefficients $\alpha _k$ are given by

2.10 $$\begin{aligned} \alpha _k =\mathbb {E}_{\tilde{\mathbb {P}}}\left[ X e_k(A_T(Z))\right] . \end{aligned}$$

As for LSMC, the representation of X in Eq. (2.9) involves infinitely many parameters, which leaves a direct estimation infeasible. Consequently, the right-hand side of Eq. (2.9) is truncated to a finite number *K*;

2.11 $$\begin{aligned} g_T^K=\sum _{k=1}^{K} \alpha _k e_k =\left( \varvec{\alpha }_K \right) ^T\varvec{e}_K, \end{aligned}$$

where $\varvec{\alpha }_K=(\alpha _1,\ldots ,\alpha _K)^T$ and $\varvec{e}_K=(e_1,\ldots ,e_K)^T$. Defining the approximation error $a_T^K$ as usual by $a_T^K:=g_T-g_T^K$ , we obtain the representation

2.12 $$\begin{aligned} X=g_T^K(A_T(Z))+a_T^K(A_T(Z)). \end{aligned}$$

This gives the regression equation for the replicating portfolio problem, where $a_T^K$ represents the regression error.^2^ Now given a (simulated) sample of size *N* denoted by $(x_1,A_T(z_1)),\ldots ,(x_N,A_T(z_N))$ , we estimate $g_{T}^K$ by least squares regression leading to

2.13 $$\begin{aligned} \hat{g}_{T}^K = \left( \varvec{\hat{\alpha }}_K\right) ^T \mathbf {e}_K, \end{aligned}$$

with

2.14 $$\begin{aligned} {\hat{\varvec{\alpha }}}_K = ((\varvec{E}_K)^T\varvec{E}_K)^{-1} (\varvec{E}_K)^T\varvec{X}, \end{aligned}$$

where $\varvec{X} = (x_{1}, \ldots , x_{N})^T$ and $\varvec{E}_K$ is an $N \times K$ matrix with the *n*t row equal to $\mathbf {e}_K(A_T(z_n))$, $n=1,\ldots ,N$. Notice that ${\hat{\varvec{\alpha }}}_K$ corresponds to the usual least squares estimator from a regression of *X* against *K* basis functions valued at time *T*. Recall that in regress-now, in contrast, *X* is regressed against basis functions valued at time *t*.

**Fig. 5:**
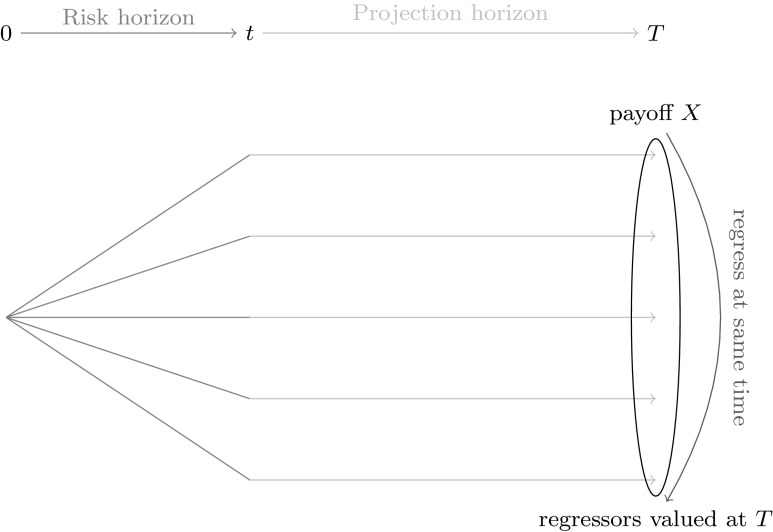
Illustration of the replicating portfolio approach

We illustrate the replicating portfolio approach in Fig. 5. Based on calibration scenarios, the replicating portfolio estimator is constructed by regressing the payoff function *X* against regressors valued at the same time point *T*. The least squares regression approach naturally provides thereby an estimate for the payoff function *X* since $\mathbb {E}_{\tilde{\mathbb {P}}}[X | \mathcal {F}_T] = X$. Given this estimate, the time *t* value of the regressors must be determined to get an estimate for the conditional expectation function $\mathbb {E}_{\tilde{\mathbb {P}}}[X | \mathcal {F}_t]$. This in turn can then be used to obtain an empirical distribution of the time *t* values at the risk horizon *t* in order to extract risk figures.

Just like the LSMC estimator, the replicating portfolio estimator is also subject to an error. However, the replicating portfolio estimator $\hat{g}_T^K$ involves only two sources of error resulting from an approximation and an estimation error. The estimation error again arises from estimating the coefficients of the regression equation based on a finite sample and converges to zero as $N \rightarrow \infty$. To better see this, we again consider the coefficient error

$$\begin{aligned} ( {\hat{\varvec{\alpha }}}_K - \varvec{\alpha }_K)&= ((\varvec{E}_K )^T\varvec{E}_K)^{-1} (\varvec{E}_K)^T (\varvec{X} - \varvec{E}_K \varvec{\beta }_K) \\&= ((\varvec{E}_K )^T\varvec{E}_K )^{-1} (\varvec{E}_K)^T ( ( \varvec{X} - \varvec{E} \varvec{\alpha } ) + (\varvec{E} \varvec{\alpha } - \varvec{E}_K \varvec{\alpha }_K ) ) \\&= ((\varvec{E}_K )^T\varvec{E}_K )^{-1} (\varvec{E}_K)^T \varvec{a}_{T}^K. \end{aligned}$$

#### Remark 2

We remark again that the functions to be approximated with LSMC and portfolio replication differ. In LSMC, we directly estimate the conditional expectation function, while in portfolio replication the approximation to the conditional expectation function is obtained by applying the conditional expectation operator to the obtained proxy of the payoff function. This also implies that the error of the time *t* value in portfolio replication is not $a_T^K(A_T(Z))$, but $\mathbb {E}_{\tilde{\mathbb {P}}}[a_T^K(A_T(Z)) | \mathcal {F}_t]$. Since the replicating portfolio is used in the Solvency II context as a proxy to the liability value in extreme scenarios, ensuring a very small error at time *t* is of utmost importance. We will return later to this point.

Compare the regression equation for LSMC (2.7) with the regression equation of the replicating portfolio (2.12). Clearly, the regression error of LSMC is composed of an approximation and a projection error, while the regression error of the replicating portfolio only contains an approximation error. Notice that for both methods the approximation error vanishes for $K \rightarrow \infty$. For the replicating portfolio, this implies that the regression error converges to zero as the number of basis functions grows. The replicating portfolio approach is thus a nonstandard regression problem. In contrast, even when the approximation error is zero, the LSMC regression error still contains the projection error. We will discuss the implications of the replicating portfolio being a nonstandard regression problem in the next section.

## Impact of the zero projection error in portfolio replication

In Sect. 2, we have outlined the Monte Carlo regression frameworks for constructing LSMC and replicating portfolio estimates. We have stressed that in LSMC the payoff function *X* at time *T* is regressed against basis functions valued at time $t < T$, while in portfolio replication it is regressed against basis functions valued at the same time point *T*. This subtle but critical distinction leads to very different characterizations of the regression problem. The regression error of the replicating portfolio method only contains an approximation error, which converges to zero in the limit as more and more basis terms are included in the representation. The LSMC regression error also contains an approximation error, which vanishes in the limit, but, due to the time gap of the regressand and the regressors, the regression error additionally contains a projection error. The difference in the composition of the regression error has several consequences that we want to illuminate throughout the subsequent sections.

### Function fitting versus portfolio replication

We have earlier pointed out that two types of Least Squares Monte Carlo approaches are discussed in the literature: LSMC regress-now, which we have referred to as LSMC in this paper, and LSMC regress-later. Also, we have indicated that LSMC Regress-Later is actually portfolio replication, and we have used this terminology throughout the paper. Now, we want to take a closer look at the reason why the least squares regression framework for replicating portfolios in Sect. 2.2 is truly a replication approach and why the least squares regression framework for LSMC in Sect. 2.1 is not.

Let us first clarify the terms “replicating portfolio” and “function fitting”. A replicating portfolio of a target claim is a portfolio of instruments that has the same properties as the target. In line with the definitions in [31, 35], we consider a replicating portfolio as a portfolio of instruments that has the same terminal cash flow as the target. By construction, we achieve this in the Hilbert space framework of Sect. 2, where the replicating portfolio of *X* is given by the infinite basis representation of Eq. (2.9). The regression equation for *X* then involves an approximation error from truncating the basis to $K < \infty$. With function fitting, we refer to the construction of a smooth function that best approximates the observed data. Least squares regression in its standard form is a data-fitting approach that focuses on finding a smooth curve that best explains the variation in observed data with random errors. Now, for both LSMC and portfolio replication, we apply the least squares regression technique. However, for LSMC, we approximate an unknown function based on noisy data, while for portfolio replication we want to find an exact representation for the (known) payoff function based on simulated data points. Thus, in LSMC we face a noisy regression, while in portfolio replication the regression is non-noisy even when the approximation error is nonzero. To better see this, we will next analyze the variance of the residuals in both LSMC and portfolio replication.

Let us consider the regression error in LSMC first, which is given by the sum of the approximation and the projection error, i.e. $a_{0,t}^K(A_t(Z)) + p_{0,t}(A_T(Z))$. For the variance of the regression error, we obtain

3.1 $$\begin{aligned}&\mathbb {V}\text {ar}\left( a_{0,t}^K(A_t(Z)) + p_{0,t}(A_T(Z))\right) \end{aligned}$$

3.2 $$\begin{aligned}&\quad = \mathbb {V}\text {ar}\left( a_{0,t}^K(A_t(Z))\right) + \mathbb {V}\text {ar}\left( p_{0,t}(A_T(Z))\right) \nonumber \\&\quad = \sum _{k=K+1}^{\infty } \beta _k^2 - \left( \mathbb {E}_{\tilde{\mathbb {P}}}[a_{0,t}^K(A_t(Z))] \right) ^2 + \mathbb {E}_{\tilde{\mathbb {P}}}[X^2] - \mathbb {E}_{\tilde{\mathbb {P}}}\left[ \left( \mathbb {E}_{\tilde{\mathbb {P}}}[X | \mathcal {F}_t] \right) ^2 \right] \nonumber \\&\quad = \sum _{k=K+1}^{\infty } \beta _k^2 - \left( \mathbb {E}_{\tilde{\mathbb {P}}}[a_{0,t}^K(A_t(Z))] \right) ^2 + \mathbb {E}_{\tilde{\mathbb {P}}}[X^2] - \sum _{k=1}^{\infty } \beta _k^2 \nonumber \\&\quad = \mathbb {E}_{\tilde{\mathbb {P}}}[X^2] - \sum _{k=1}^{K} \beta _k^2 - \left( \mathbb {E}_{\tilde{\mathbb {P}}}[a_{0,t}^K(A_t(Z))] \right) ^2, \end{aligned}$$

where we have exploited that

$$\begin{aligned} \mathbb {E}_{\tilde{\mathbb {P}}}[p_{0,t}(A_T(Z)) v_k(A_t(Z))] = 0 \; \forall k. \end{aligned}$$

Notice that, as the approximation error vanishes for $K \rightarrow \infty$ , the variance of the regression error converges to the variance of the projection error, i.e.

3.3 $$\begin{aligned} \mathbb {V}\text {ar}\left( p_{0,t}(A_T(Z)) \right)&= \mathbb {E}_{\tilde{\mathbb {P}}}\left[ \left( p_{0,t}(A_T(Z)) \right) ^2 \right] \nonumber \\&= \mathbb {E}_{\tilde{\mathbb {P}}}[X^2] - \mathbb {E}_{\tilde{\mathbb {P}}} \left[ \left( \mathbb {E}_{\tilde{\mathbb {P}}}[X | \mathcal {F}_t]\right) ^2 \right] \end{aligned}$$

3.4 $$\begin{aligned}&= \mathbb {E}_{\tilde{\mathbb {P}}}[X^2] - \sum _{k=1}^{\infty } \beta _k^2. \end{aligned}$$

Since we know that *X* is expressible in terms of an infinite orthonormal basis, i.e. $X = \sum _{k=1}^{\infty } \alpha _k e_k(A_T(Z))$, we can even write

3.5 $$\begin{aligned} \mathbb {V}\text {ar}\left( p_{0,t}(A_T(Z)) \right) = \sum _{j=1}^{\infty } \alpha _j^2 - \sum _{k=1}^{\infty } \beta _k^2. \end{aligned}$$

We also want to investigate the conditional variance of the regression error:

3.6 $$\begin{aligned}&\mathbb {V}\text {ar}\left( a_{0,t}^K(A_t(Z)) + p_{0,t}(A_T(Z)) | \mathcal {F}_t \right) \nonumber \\&\quad = \mathbb {V}\text {ar}\left( a_{0,t}^K(A_t(Z)) | \mathcal {F}_t\right) + \mathbb {V}\text {ar}\left( p_{0,t}(A_T(Z)) | \mathcal {F}_t \right) \nonumber \\&\quad \quad + 2 \; \text {Cov} \left( a_{0,t}^K(A_t(Z)), p_{0,t}(A_T(Z)) | \mathcal {F}_t \right) \nonumber \\&\quad = \mathbb {E}_{\tilde{\mathbb {P}}} \left[ \left( p_{0,t}(A_T(Z)) \right) ^2 | \mathcal {F}_t \right] \nonumber \\&\quad = \mathbb {V}\text {ar}\left[ X | \mathcal {F}_t \right] . \end{aligned}$$

This is the conditional variance of the target function *X*. Depending on the underlying stochastic processes and the structure of *X*, it may well be that the conditional variance of the time *T* random payoff *X* varies with observations at time *t*. Therefore, in LSMC, we may potentially deal with heteroskedastic residuals.

We repeat the analysis of the variance of the regression error for the replicating portfolio approach. Recall that the regression error in portfolio replication is given by $a_T^K(A_T(Z))$. For the variance, we obtain

3.7 $$\begin{aligned} \mathbb {V}\text {ar}\left( a_T^K(A_T(Z)) \right)&= \mathbb {E}_{\tilde{\mathbb {P}}}\left[ \left( a_T^K(A_T(Z)) \right) ^2 \right] - \left( \mathbb {E}_{\tilde{\mathbb {P}}}[a_T^K(A_T(Z))] \right) ^2 \nonumber \\&= \sum _{k=K+1}^{\infty } \alpha _k^2 - \left( \mathbb {E}_{\tilde{\mathbb {P}}}[a_T^K(A_T(Z))] \right) ^2. \end{aligned}$$

Clearly, the variance converges to zero in the limit for $K \rightarrow \infty$ as the perfect replicating portfolio is attained. Let us take a look at the conditional variance of the residual of the replicating portfolio problem:

3.8 $$\begin{aligned} \mathbb {V}\text {ar}\left( a_T^K(A_T(Z)) | \mathcal {F}_T \right)&= 0. \end{aligned}$$

The zero conditional variance of the residuals implies that there is no variation of the error at each observation of $A_T(Z)$. This actually makes sense, as the residual simply reflects the approximation error, which is clearly defined at each observation of $A_T(Z)$. We can therefore understand the replicating portfolio approach as non-noisy even when the approximation error is nonzero. Summing up, in portfolio replication, the conditional variance of the residuals is zero and the unconditional variance of the residuals converges to zero as the number of basis terms grows. Thus, the perfect replicating portfolio is attained that truly reproduces the terminal payoff *X*. Consequently, the least squares regression approach underlying the replicating portfolio approach is not a typical regression approach of fitting a function through a cloud of data. In the following, we give two simple examples which illustrate the nonstandard regression problem in portfolio replication and the noisy regression problem in LSMC.

#### Example 1

(Simple Brownian motion)

Let us consider the most simple example, where the approximation errors are zero for LSMC and portfolio replication. The payoff function is given by $X= W_T$ with $W_T$ being a standard Brownian motion. As regressors, we take $W_t$ for LSMC and $W_T$ for portfolio replication. Obviously, for portfolio replication, a perfect fit is achieved. Consequently, the conditional expectation function $g_t(W_t) = W_t$ is also perfectly fit for any $t \le T$. For LSMC, the approximation error is zero, but we are still faced with a noisy regression due to the persistence of the projection error. The projection error is

$$\begin{aligned} p_{0,t}(W_T) = X - \mathbb {E}[X | \mathcal {F}_t] = W_T - W_t. \end{aligned}$$

As Brownian motions have stationary independent increments, the distribution of $(W_T-W_t)$ is independent of information at time *t*. Therefore, we have

$$\begin{aligned} \mathbb {V}\text {ar}(W_T-W_t)&= \mathbb {V}\text {ar}(W_T-W_t | \mathcal {F}_t) \\&= T-t. \end{aligned}$$

**Fig. 6:**
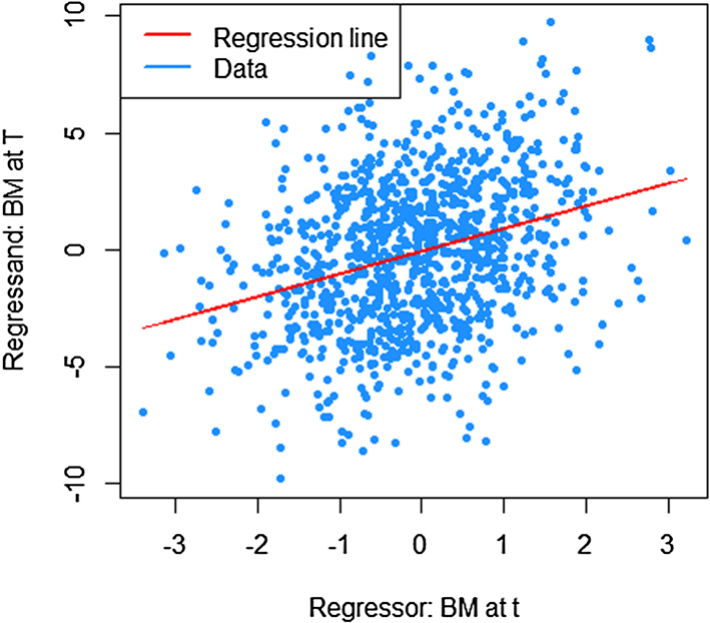
Noisy regression in LSMC (Example 1)

**Fig. 7:**
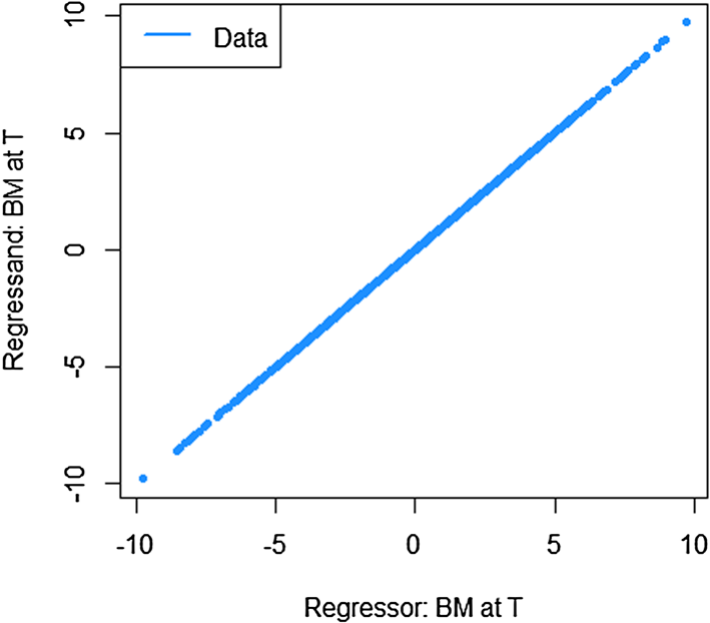
Regression in portfolio replication (Example 1)

We illustrate this in Figs. 6 and 7, where we have plotted the LSMC and the portfolio replication regression problem for the simple Brownian motion example with $t=1$ and $T=10$. Figure 6 gives the LSMC regression problem by plotting the regressand $W_T$ against the regressor $W_t$. Least squares regression of $W_T$ on $W_t$ returns the function that best fits the cloud of data. By construction, the best line is the conditional expectation $\mathbb {E}_{\tilde{\mathbb {P}}}[W_T | W_t]$.

#### Example 2

(Exponential function)

We take a simple exponential function to be replicated

$$\begin{aligned} X = e^{\sigma W_T} \end{aligned}$$

with $W_T$ a standard Brownian motion. The conditional expectation is then

$$\begin{aligned} \mathbb {E}[X | \mathcal {F}_t] = e^{\sigma W_t + \frac{1}{2} \sigma ^2 (T-t)}. \end{aligned}$$

We investigate the following LSMC and portfolio replication regression equations

$$\begin{aligned}&X = \beta _0 + \beta _1 W_t + \epsilon _t \\&X = \alpha _0 + \alpha _1 W_T + \epsilon _T. \end{aligned}$$

**Fig. 8:**
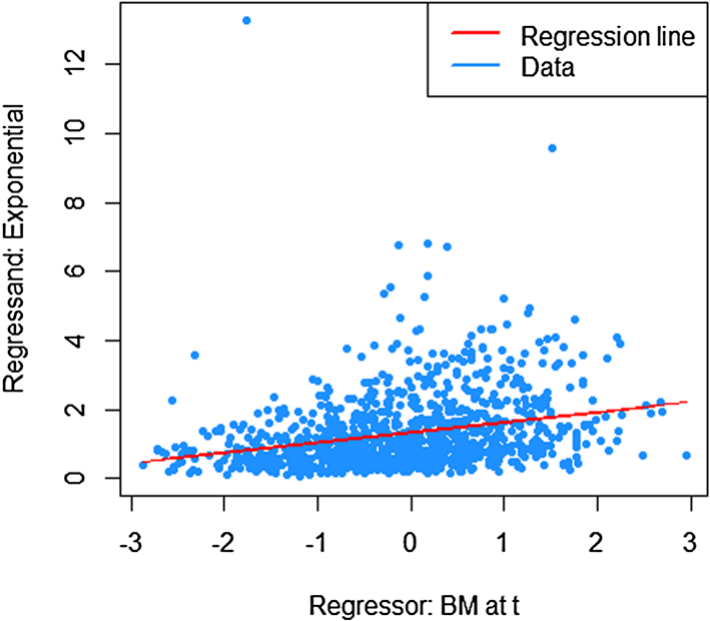
Noisy regression in LSMC (Example 2)

**Fig. 9:**
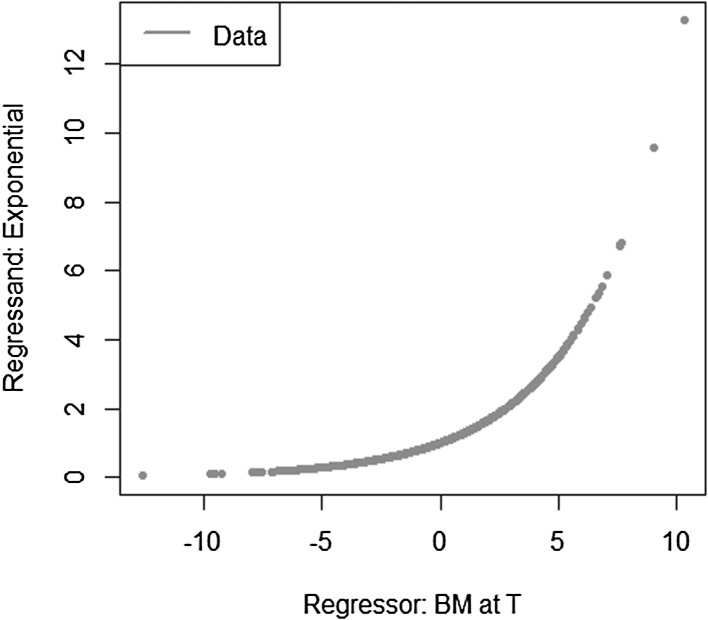
Regression in portfolio replication (Example 2)

Recall that for LSMC the regression error $\epsilon _t$ consists of an approximation and a projection error, while for the replicating portfolio problem the nonstandard regression error $\epsilon _T$ involves only an approximation error. We can clearly see this from Figs. 8 and 9. For the example at hand, the LSMC regression problem is heteroskedastic. Even if the approximation error was zero in LSMC, the projection error persists and the noisy regression would still be heteroskedastic. To see this, consider the conditional variance of the projection error

$$\begin{aligned} \mathbb {V}\text {ar}(p_{0,t}(W_T) | \mathcal {F}_t)&= \mathbb {E}[e^{2\sigma W_T} | \mathcal {F}_t ] - e^{2 \sigma W_t + \sigma ^2 (T-t)} \\&= e^{2 \sigma W_t + \sigma ^2 (T-t)} ( e^{\sigma ^2 (T-t)} -1 ), \end{aligned}$$

which clearly increases for larger values of the Brownian motion at time *t*.

#### Example 3

(Artificial portfolio with perfect basis)

In this example, we construct a portfolio of puts and calls in the Black–Scholes framework. As basis, we use the components that make up the payoff function, which ensures that at least theoretically the perfect representation for both portfolio replication and LSMC is available. Let *X* be as defined below

3.9 $$\begin{aligned} X&= 100- 2(K_1-S(T))^{+} + (S(T)-K_2)^{+} - 2 (S(T)-K_3)^{+} + (S(T)-K_4)^{+} \nonumber \\&\quad + 0.5 (S(T)-K_5)^{+} -0.5 (S(T)-K_6)^{+} \end{aligned}$$

with strikes $K_i = S_0 e^{(\mu -\frac{1}{2} \sigma ^2) T + \sigma \sqrt{T} z_i}$ where $\lbrace z_i \rbrace _{i=1}^6=\lbrace -1.5,-0.5,0,1,1.5,2 \rbrace$. The parameters are defined in Table 1, where *r* is the risk-free rate, *N* is the sample size of the calibration set and *m* is the sample size for the out-of-sample set.

**Table 1:** Parameters for Example 12

*t*	*T*	$\mu$	$\sigma$	*r*	$S_0$	*N*	*m*
1	5	0.08	0.2	0.02	100	1200	5000

Ultimately, we want to find an approximation to the price of *X* at time *t*. We estimate the replicating portfolio by regressing the values of *X* against the basis and price the basis using the Black–Scholes formula in order to obtain the pricing function at time *t*. With LSMC, an estimate of the pricing function at time *t* is obtained directly by regressing the discounted payoff *X* against the time *t*-prices of the basis. The calibration sample set is based on the risk-neutral measure here. We will come back to the relevance of the measure in Sect. 3.4. As the correct price of the target function *X* is available in the Black–Scholes framework the LSMC and portfolio replication results can be assessed against it.

The optimal solution for the coefficients of the LSMC and replicating portfolio representation is

3.10 $$\begin{aligned} \varvec{\alpha } = \varvec{\beta } = \left( 100,-2,1,-2,1,0.5,-0.5 \right) ^T. \end{aligned}$$

**Fig. 10:**
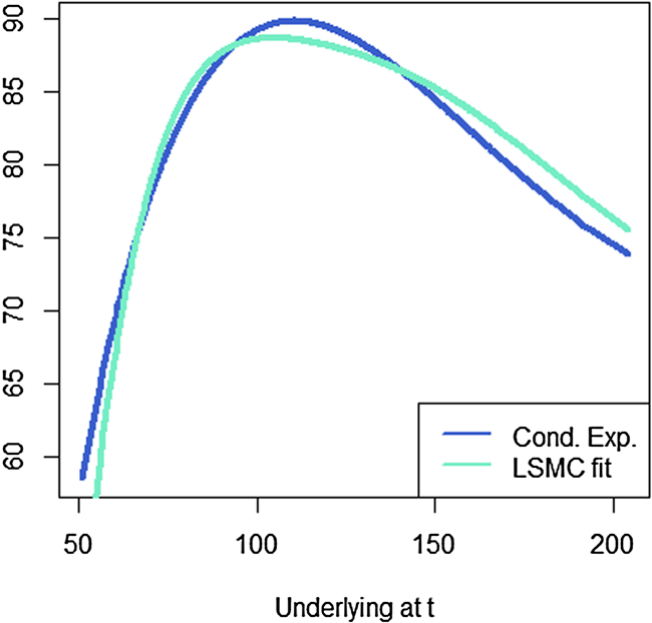
LSMC fit for $N=1200$ (Example 3)

**Fig. 11:**
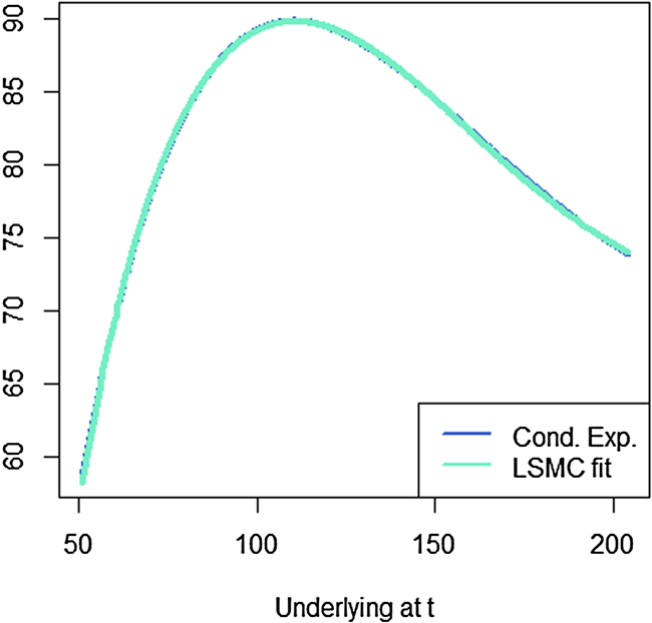
LSMC fit for $N=1,000,000$ (Example 3)

When estimating the replicating portfolio on a sufficiently diverse scenario set, exactly these coefficients are obtained. Also given the perfect replicating portfolio, the conditional expectation at any $t<T$ is perfectly obtained by pricing the basis terms. For LSMC,we do not get the exact result for the coefficients although the perfect basis is available. On a sample with size $N=1200$. Figure 10 illustrates the imperfect fit that results. With sample size $N=1,000,000$ , the conditional expectation function is very well fitted with an $R^2$ of $99.99\, \%$ (see Fig. 11). The estimated coefficients, though, are

3.11 $$\begin{aligned} \hat{\varvec{\beta }} = ( 101.82,-2.10,0.19,-0.15,-4.12,8.65,-5.25)^T \end{aligned}$$

and thus differ from the coefficients that would return the replicating portfolio. Clearly, LSMC is a function-fitting method and not a portfolio replication method.

#### Example 4

(Equity swap)

In this example we consider a simple equity swap with payoff at maturity *T*

$$\begin{aligned} X = S_2(T)-S_1(T), \end{aligned}$$

where $S_1(T)$ and $S_2(T)$ are modeled as uncorrelated geometric Brownian motions

$$\begin{aligned} S_i(T) = S_i(0) e^{\left( \mu _i-\frac{1}{2} \sigma _i^2 \right) T + \sigma _i W(T)}, \quad i=1,2 \end{aligned}$$

with parameters $\mu _1=0.08$, $\sigma _1=0.2$, $\mu _2=0.05$ and $\sigma _2=0.15$. The payoff *X* depends on the values of both assets $S_1(T)$ and $S_2(T)$. Its conditional expectation function at time *t* also requires the information of both assets at time *t*, $S_1(t)$ and $S_2(t)$. Let us now consider the construction of both replicating portfolio and LSMC estimates, where the risk factors are not correctly identified. In other words, the regression equation misses regressors constructed on relevant risk factors. The regression functions are specified for portfolio replication and LSMC, respectively as,

$$\begin{aligned}&X = \alpha _0 + \alpha _1 S_1(T) + \epsilon _T \\&X = \beta _0 + \beta _1 S_1(t) + \epsilon _t. \end{aligned}$$

**Fig. 12:**
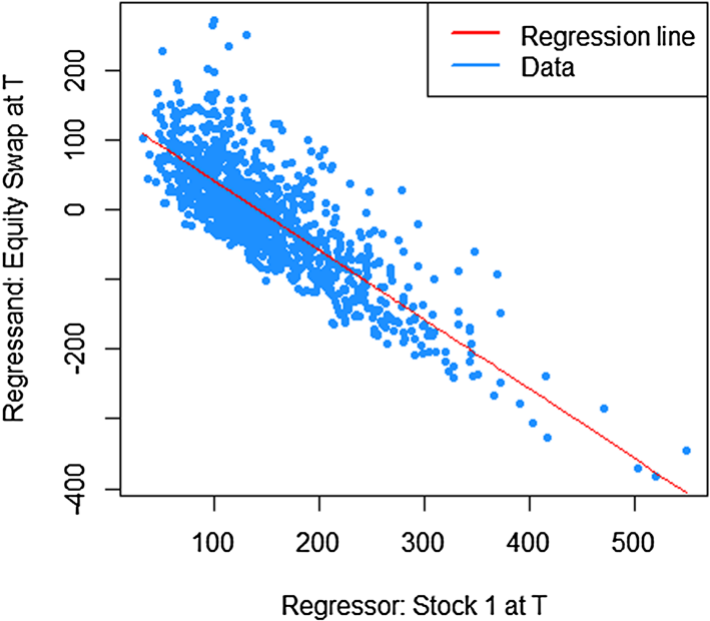
Missing risk factors regression in portfolio replication (Example 4)

**Fig. 13:**
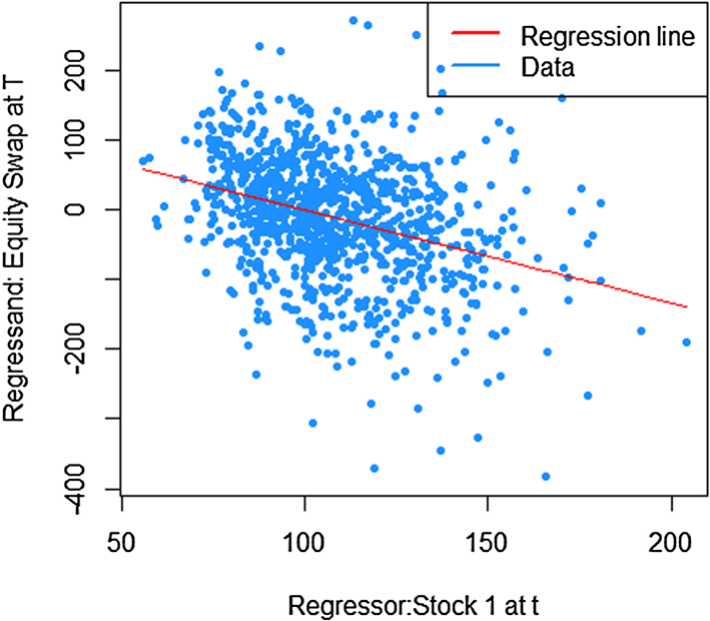
Missing risk factors regression in LSMC (Example 4)

Figures 12 and 13 illustrate the regression of the payoff function *X* against $S_1(T)$ in portfolio replication and $S_1(t)$ in LSMC. Both figures reveal noisy regressions. While for LSMC a noisy regression is not surprising, for portfolio replication this is not expected if all risk factors have been correctly identified. Consequently, risk factors must have been neglected in the replicating portfolio. Note that for LSMC this conclusion cannot be drawn as the regressions are always noisy.

Regressing only against $S_1(t)$ still yields an estimated conditional expectation function, i.e. the expectation conditional on the smaller information set $S_1(t)$, but this is not the conditional expectation function of interest. For the replicating portfolio missing the information of $S_2(T)$ , the resulting $R^2$ is $66.75 \,\%$. For the LSMC regression, it is $11.21 \,\%$. The details on $R^2$ as a measure for the goodness of fit of both portfolio replication and LSMC will be explained in Sect. 3.2. Nonetheless, it is worthwhile to mention at this point that in LSMC it is usual to observe a low $R^2$. In portfolio replication, in contrast, a low $R^2$ either signifies a large approximation error, i.e. a larger number of basis functions is required to obtain a better replicating portfolio,^3^ or, risk factors are missing, i.e. $A_T(Z)$ is not correctly identified.

The last example has shown that with the LSMC approach a conditional expectation is always estimated, it may just not be the one in which we are actually interested. Due to the time gap of the regressand and the regressors, the LSMC regression is noisy by construction. Detecting the issue of potentially having neglected relevant risk factors is therefore difficult. For portfolio replication, the regression is not noisy given that all underlying risk factors of the payoff function have been identified. $R^2$ is a useful measure that provides important information on the approximation error of the regression in portfolio replication (see Sect. 3.2). A low $R^2$ may moreover be an indicator for missing risk factors.

So far, we have delivered the argument that LSMC is a function-fitting approach as its least squares regression is noisy. The least squares approach to portfolio replication is, in contrast, nonstandard as the regression error converges to zero in the limit and the conditional variance of the residuals is zero. In that context, there is one more argument why the least squares approach of Sect. 2.2 is truly a replicating portfolio approach while the least squares approach of Sect. 2.1 is not. In portfolio replication, the payoff function at time *T* is approximated. The conditional expectation function at any $t < T$ is then obtained by calculating the time *t* value of the basis terms that make up the approximation of the target payoff function *X*. The better the replicating portfolio mirrors the payoff function at time *T*, the better the fit to the conditional expectation functions at any time $t < T$. Straightforwardly, this implicates a great amount of flexibility, particularly if the conditional expectation at several time points is of interest. With LSMC, in contrast, the conditional expectation at a particular $t^{*} < T$ is approximated by regressing basis terms valued at time $t^{*}$ against the target payoff function *X* valued at time *T*. The result is an approximation of the conditional expectation at the particular time point $t^{*}$ and does not necessarily imply an approximation of the conditional expectations at times $t<T$ with $t \ne t^{*}$. Consider the representations for *X* and $\mathbb {E}_{\tilde{\mathbb {P}}}[X | \mathcal {F}_t]$

$$\begin{aligned}&g_T(A_T(Z)) = \sum _{k=1}^{\infty } \alpha _k e_k(A_T(Z)) \\&g_{0,t}(A_t(Z)) = \sum _{k=1}^{\infty } \beta _k v_k(A_t(Z)). \end{aligned}$$

Moreover,

$$\begin{aligned} g_T(A_T(Z)) = g_{0,t}(A_t(Z)) + p_{0,t}(A_T(Z)). \end{aligned}$$

Given the replicating portfolio of *X*, we obtain $\mathbb {E}_{\tilde{\mathbb {P}}}[X|\mathcal {F}_t]$ for any $t < T$ by taking the conditional expectation of the basis terms, i.e.

$$\begin{aligned} \mathbb {E}_{\tilde{\mathbb {P}}}[X | \mathcal {F}_t] = \sum _{k=1}^{\infty } \alpha _k \mathbb {E}_{\tilde{\mathbb {P}}}[e_k(A_T(Z)) | \mathcal {F}_t]. \end{aligned}$$

For the LSMC representation of the conditional expectation at a particular time point $t^{*} < T$, $g_{0,t^{*}}(A_{t^{*}}(Z))=\sum _{k=1}^{\infty } \beta _k v_k(A_{t^{*}}(Z))$, the same holds for $t <t^{*}$ only if we can compute the conditional expectations of the basis terms and the projection error, i.e.

$$\begin{aligned} \mathbb {E}_{\tilde{\mathbb {P}}}[X | \mathcal {F}_t] = \sum _{k=1}^{\infty } \beta _k \mathbb {E}_{\tilde{\mathbb {P}}}[v_k(A_{t^{*}}(Z)) | \mathcal {F}_t] + \mathbb {E}_{\tilde{\mathbb {P}}}[p_{0,t^{*}}(A_T(Z)) | \mathcal {F}_t], \quad t < t^{*}. \end{aligned}$$

It is to be expected that the calculation of the conditional expectation of the projection error is most likely not straightforward, particularly when considering that LSMC is used in applications, for which already the time $$t^{*}$$ conditional expectation is not closed-form available. In order to get $$\mathbb {E}_{\tilde{\mathbb {P}}}[X | \mathcal {F}_t]$$ for $$ t^{*}< t < T$$ $$g_{0,t^{*}}(A_{t^{*}}(Z))$$ must be corrected by the time *t* conditional expectation of the projection error

$$\begin{aligned} \mathbb {E}_{\tilde{\mathbb {P}}}[X | \mathcal {F}_t]&= g_{0,t^{*}}(A_{t^{*}}(Z)) + \mathbb {E}_{\tilde{\mathbb {P}}}[p_{0,t^{*}}(A_T(Z)) | \mathcal {F}_t], \; t^{*}< t < T\\&= g_{0,t^{*}}(A_{t^{*}}(Z)) + \left( \mathbb {E}_{\tilde{\mathbb {P}}}[X | \mathcal {F}_t] - \mathbb {E}_{\tilde{\mathbb {P}}}[X | \mathcal {F}_{t^{*}}] \right) , \end{aligned}$$

where again the calculation of the conditional expectation of the projection errors is probably not straightforward. Moreover, it cannot simply be inferred that the LSMC representation at time $t^{*}$ also holds at time *t*, $t > t^{*}$, by valuing the basis at time *t*. Thus given the time $t^{*}$ coefficients $\lbrace \beta _{k,t^{*}} \rbrace _{k=1}^{\infty }$, which we denote with the subscript $t^{*}$, it cannot be inferred that

$$\begin{aligned} \mathbb {E}_{\tilde{\mathbb {P}}}[X | \mathcal {F}_t] = \sum _{k=1}^{\infty } \beta _{k,t^{*}} v_k(A_t(Z)). \end{aligned}$$

#### Example 5

(*Example* 3 *revisited: Artificial portfolio with perfect basis*)

**Fig. 14:**
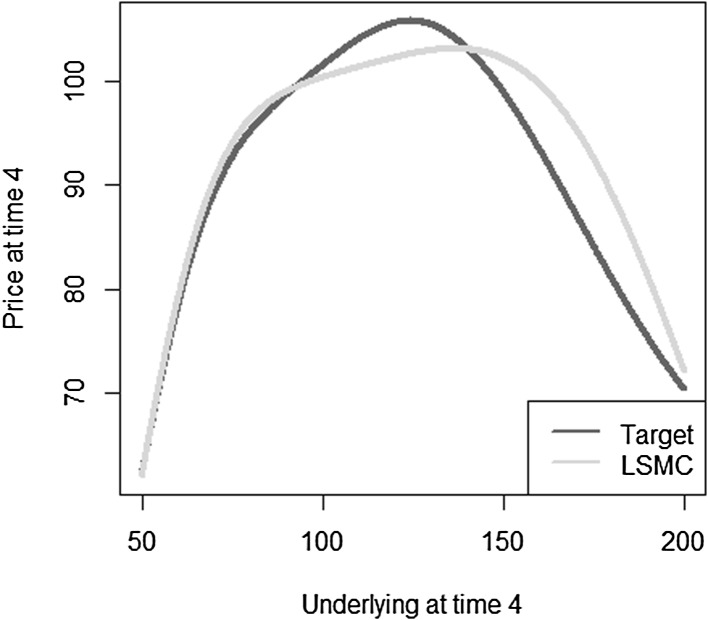
Illustration of LSMC fit at $t=4$ with calibration at time 1 (Example 5)

Reconsider Example 3, for which a very good fit to the conditional expectation $\mathbb {E}[X | \mathcal {F}_1]$ has been found with LSMC. Using the estimated coefficients in (3.11) and the prices of the basis at time $t=4$ , the resulting fit to the conditional expectation at time $t=4$ is assessed. Figure 14 highlights that the LSMC coefficients calibrated to the conditional expectation at time 1 do not imply a good fit to the conditional expectation at a different time point. This is in contrast to a portfolio replication approach. Remember that with portfolio replication the correct coefficients as in (3.10) have been identified. Thus, automatically, the conditional expectation for any $t < T$ is also perfectly obtained by applying the conditional expectation operator to the replicating portfolio.

#### Example 6

(LSMC with Hermite polynomials)

**Fig. 15:**
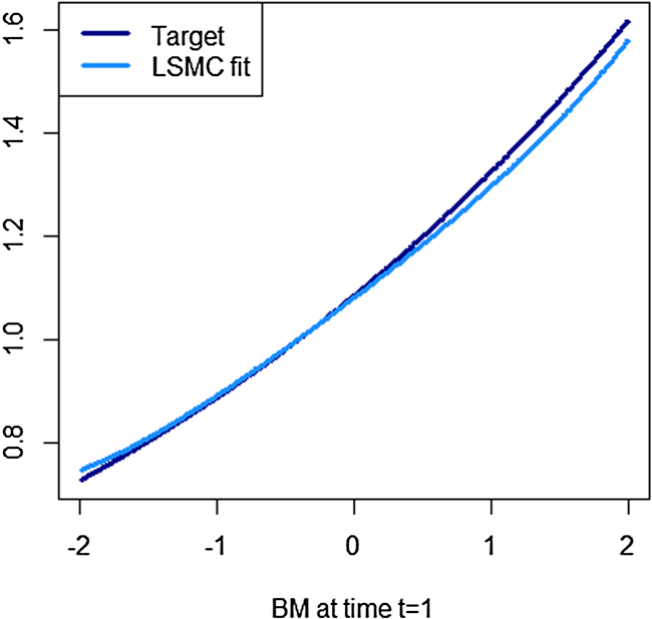
LSMC fit at $t^{*}=1$ (Example 6)

**Fig. 16:**
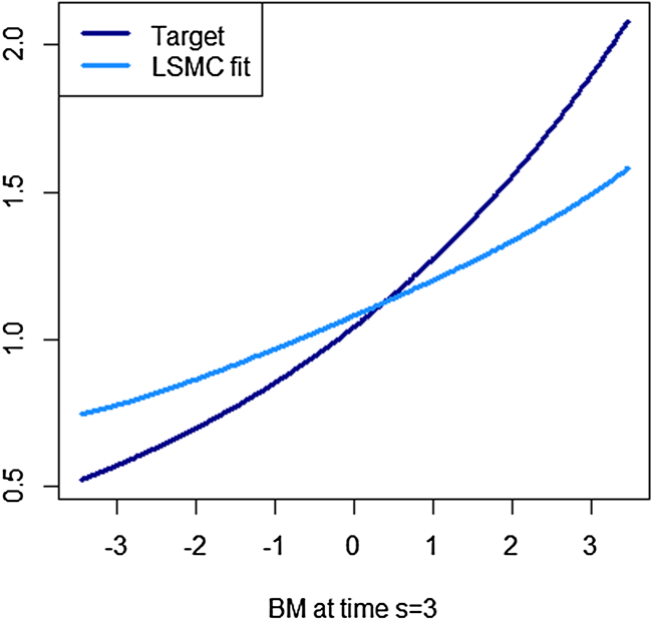
LSMC fit at $t=3$ given calibration at $t^{*}$ (Example 6)

The simple exponential payoff function from Example 2 is taken, for which the LSMC technique with a basis of Hermite polynomials is applied to approximate its conditional expectation function. Let $T=5$ and $\sigma =0.2$. We simulate 1000 paths of a Brownian motion, $\lbrace W_{t^{*}}, W_T \rbrace$ with $t^{*}=1$, and consider the Hermite polynomials on $\left( W_{t^{*}} \slash \sqrt{t^{*}} \right)$. With only $K=5$ Hermite terms a reasonably good fit is achieved, which is visualized in Figure 15. However, taking the coefficients from the time $t^{*}=1$ calibration and valuing the Hermite polynomials at a different time point *t*, $t^{*}< t <T$, does not yield a good representation for the conditional expectation function at time *t*. Figure 16 illustrates this for $t=3$. The example indicates that a good representation of the conditional expectation at a particular time point does not imply a similarly good representation of the conditional expectation at a different time point.

Summarizing, we can generally infer the following. The least squares Monte Carlo approach of Sect. 2.2 is truly a replicating portfolio approach for the subsequent reasons.

- The better the replicating portfolio approximates the target payoff function, the better the resulting fit to the conditional expectation function at any time *t*, $t < T$. A perfect replicating portfolio thus implies a perfect fit to the conditional expectation function at any time *t*, $t < T$.
- This is linked to the fact that the least squares Monte Carlo approach of Sect. 2.2 is nonstandard resulting in a non-noisy regression.

LSMC is a function-fitting method where the estimation of the conditional expectation function at a specific time point is attained by fitting a curve through noisy data. The LSMC representation of the conditional expectation at a distinct time point does not straightforwardly suggest a representation to the conditional expectation at a different time point.

### Upper limit of *R*-square: LSMC versus portfolio replication

In this section, we want to discuss the squared correlation coefficient $R^2$ as a measure for the goodness of fit. We will explain that the measure $R^2$, which is typically calculated in a least squares regression, is not a useful measure in LSMC, but is meaningful for portfolio replication. In fact, the $R^2$ in LSMC can never be 1 even if the conditional expectation function is perfectly fitted, unless $t = T$, which would not correspond to LSMC, but to portfolio replication. Intuitively, we expect the target function *X* at time *T* to correlate more strongly with regressors valued at time *T* than with regressors valued at time *t*. This is the more pronounced the greater the gap between the time points *t* and *T*. Thus, the observation that $R^2$ is not meaningful for LSMC is caused by the persistence of the projection error in LSMC.

In a first step, and to illustrate our result, we now assume that the approximation error is zero, i.e. we compare the LSMC and portfolio replication result with zero approximation error. From the LSMC regression equation, we then have

$$\begin{aligned} X = \mathbb {E}_{\tilde{\mathbb {P}}}[X | \mathcal {F}_t] + p_{0,t}(A_T(Z)) \end{aligned}$$

with $p_{0,t}$ the projection error. $R^2$ is defined as the explained sum of squares (SSE) over the total sum of squares (SST), which can be written as

$$\begin{aligned} R^2 = \frac{\text {SSE}}{\text {SST}} = 1 - \frac{\text {SSR}}{\text {SST}}, \end{aligned}$$

where SSR is the residual sum of squares. By construction for portfolio replication with zero approximation error, the $R^2$ is 1 as the residual sum of squares is zero. For portfolio replication, this means in general that the higher the $R^2$ the smaller the approximation error and the closer the portfolio replication estimator is to the true result. For LSMC, we get the following $R^2$

3.12 $$\begin{aligned} R^2_{\textit{lsmc}}&= \frac{ \mathbb {E}_{\tilde{\mathbb {P}}}[(\mathbb {E}_{\tilde{\mathbb {P}}}[X | \mathcal {F}_t] - E_{\tilde{\mathbb {P}}}[X] )^2 ] }{\mathbb {E}_{\tilde{\mathbb {P}}}[ (X - \mathbb {E}_{\tilde{\mathbb {P}}}[X] )^2 ]} \nonumber \\&= 1 - \frac{\mathbb {E}_{\tilde{\mathbb {P}}}[( X - \mathbb {E}_{\tilde{\mathbb {P}}}[X | \mathcal {F}_t])^2 ]}{\mathbb {E}_{\tilde{\mathbb {P}}}[ (X - \mathbb {E}_{\tilde{\mathbb {P}}}[X] )^2 ]} \nonumber \\&= 1 - \frac{\mathbb {V}\text {ar}(p_{0,t}(A_T(Z)))}{\mathbb {V}\text {ar}(X)} \nonumber \\&= 1 - \frac{\sum _{j=1}^{\infty } \alpha _j - \sum _{k=1}^{\infty } \beta _k^2}{\mathbb {V}\text {ar}(X)}, \end{aligned}$$

which is only equal to 1 if the projection error is zero or equivalently $\mathbb {E}_{\tilde{\mathbb {P}}}[X | \mathcal {F}_t] = X$. By the definition of *X* and for $t < T$ , this is not the case. Also note that Equation (3.12) is the upper bound for the $R^2$ that can be maximally attained in LSMC. It gives the $R^2$ when only the projection error remains as regression error, meaning that the approximation error is zero and a perfect fit to the conditional expectation function $g_{0,t}$ is achieved.

#### Example 7

(Simple Brownian motion)

We again illustrate the result with the most simple example, where the approximation errors are zero for both LSMC and portfolio replication. We take the same set-up as in Example 1. Obviously, for the replicating portfolio, a perfect fit is achieved with an $R^2$ of 1. Consequently, the conditional expectation function $g_t(W_t) = W_t$ is also perfectly fit for any $t < T$. For LSMC, the goodness of fit depends on the projection error, which is driven by the time gap between *t* and *T*. This can be directly seen from the $R^2$, which is given by

$$\begin{aligned} R^2_{\text {lsmc}} = 1 - \frac{(T-t)}{T} = \frac{t}{T}. \end{aligned}$$

This is the highest $R^2$ that can be reached with the LSMC method of approximating the conditional expectation function $W_t$ through regression of $W_T$ on a basis valued at *t*.

Let us now explore the more general case, in which we allow a nonzero approximation error in both LSMC and portfolio replication. For LSMC, we obtain the following $R^2$

3.13 $$\begin{aligned} R_{\text {lsmc}}^2&= 1 - \frac{\mathbb {E}_{\tilde{\mathbb {P}}}\left[ \left( a_{0,t}^K(A_t(Z)) + p_{0,t}(A_T(Z) \right) ^2\right] }{\mathbb {V}\text {ar}(X)} \nonumber \\&= 1- \frac{\mathbb {E}_{\tilde{\mathbb {P}}}\left[ \left( a_{0,t}^K(A_t(Z))\right) ^2 \right] + \mathbb {E}_{\tilde{\mathbb {P}}} \left[ \left( p_{0,t}(A_T(Z))\right) ^2\right] }{\mathbb {V}\text {ar}(X)} \nonumber \\&= 1- \frac{\sum _{k=K+1}^{\infty }\beta _k^2 + \mathbb {E}_{\tilde{\mathbb {P}}}[X^2] - \sum _{k=1}^{\infty } \beta _k^2}{\mathbb {V}\text {ar}(X)} \nonumber \\&= 1- \frac{\sum _{j=1}^{\infty } \alpha _j^2 - \sum _{k=1}^{K} \beta _k^2}{\mathbb {V}\text {ar}(X)}, \end{aligned}$$

which is smaller than the $R^2$ of (3.12) unless $K \rightarrow \infty$, confirming again that (3.12) is the upper limit for $R^2$ in LSMC. For the $R^2$ of the least squares regression in portfolio replication, we obtain

3.14 $$\begin{aligned} R^2_{RP}&= 1 - \frac{\mathbb {E}_{\tilde{\mathbb {P}}} \left[ \left( a_T^K(A_T(Z)) \right) ^2 \right] }{\mathbb {V}\text {ar}(X)} \nonumber \\&= 1 - \frac{\sum _{k=K+1}^{\infty } \alpha _k^2}{\mathbb {V}\text {ar}(X)}. \end{aligned}$$

Clearly, the smaller the sum $\sum _{k=K+1}^{\infty } \alpha _k^2$ the higher the $R^2$ for portfolio replication. Since that sum is driven by the approximation error, we see a direct link between the $R^2$ and the approximation error and can conclude that a higher $R^2$ indicates a smaller approximation error.

#### Example 8

(Exponential function)

For Example 2, an approximation error is present in both LSMC and portfolio replication. Based on a sample $N=1000$ , we obtain for the LSMC regression an $R^2$ of 0.077, while for the replicating portfolio we obtain an $R^2$ of 0.74. If we calculate the (in-sample) mean square error for the fit of both methods to the conditional expectation function $\exp \left( \sigma W_t + \frac{1}{2} \sigma ^2 (T-t) \right)$ , we obtain comparable results with 0.004. From that we can deduce that, while both methods yield a similar quality in terms of the goodness of fit to the conditional expectation function, the $R^2$ for LSMC does not reveal this and is misleading.

Note that calculating the upper $R^2$ limit in LSMC in (3.12) for a particular target function *X* involves the calculation of the variance of the projection error. For the applications for which proxy methods such as portfolio replication and LSMC are used, we do not expect that the variance of the projection error to be readily available. Without the upper limit, judging an $R^2$ obtained for an LSMC representation becomes difficult. The $R^2$ thus does not provide information on how good or bad is the estimated representation. Drawing conclusions on missing risk factors and/or basis terms is not straightforward. This is different for portfolio replication, where the upper limit of $R^2$ is always 1 indicating a perfect fit. Consequently, we can use $R^2$ as a simple but very effective measure for assessing the quality of a replicating portfolio. Due to the direct link between $R^2$ and the approximation error $a_T^K$, we can say that the higher the $R^2$ the smaller the approximation error. Recall that in portfolio replication we have to evaluate the conditional expectation function in a second step by applying the conditional expectation operator to the replicating portfolio. The resulting error in the replicating portfolio proxy to the conditional expectation function $g_{0,t}$ is then $\mathbb {E}_{\tilde{\mathbb {P}}}[a_T^K(A_T(Z)) | \mathcal {F}_t]$. By ensuring that $a_T^K$ is small, we also ensure that $\mathbb {E}_{\tilde{\mathbb {P}}}[a_T^K(A_T(Z)) | \mathcal {F}_t]$ is small. In that respect, we can apply $R^2$ in portfolio replication as a warning signal for the quality of our proxy, i.e. only replicating portfolios with very high $R^2$ should be used. As we have seen in this section $R^2$ cannot be interpreted in the same way in LSMC.

### Asymptotic covariance with fixed truncation parameter

Intuitively we expect basis functions valued at time *T* to be more strongly correlated with the target function *X*, which is also valued at time *T*. In contrast to that we expect basis functions valued at time $t < T$ to be less strongly correlated with the target function valued at time *T*. We have first addressed this in Sect. 3.1 where we have highlighted that in LSMC we deal with noisy regressions due to the time gap in the regressand and the regressors. The analysis of $R^2$ in Sect. 3.2 furthermore confirms the hypothesis. In this section, we derive the asymptotic covariance matrix for LSMC and portfolio replication for a fixed truncation parameter *K*. Given a fixed *K*, the asymptotic distribution of ${\hat{\varvec{\alpha }}}_K$ and ${\hat{\varvec{\beta }}}_K$, respectively, is derived. Assume that the sampling schemes $((X_1,A_t(Z_1)),\ldots ,(X_N,A_t(Z_N)))$ and $((X_1,A_T(Z_1)),\ldots ,(X_N,A_T(Z_N)))$ are such that

3.15 $$\begin{aligned}&\frac{1}{N}\left( \left( \varvec{V}_K\right) ^T\varvec{V}_K\right) \mathop {\rightarrow }\limits ^{\tilde{\mathbb {P}}} C_{{{\text {lsmc}}}} \text{ and } \nonumber \\&\frac{1}{\sqrt{N}} \sum _{i=1}^N \mathbf {v}_K(A_t(Z_i))\left( a_{0,t}^K(A_t(Z_i))+p_{0,t}(A_T(Z_i))\right) \mathop {\rightarrow }\limits ^{d} N\left( 0,\varvec{\Sigma }_{\text {lsmc}}\right) \end{aligned}$$

and

3.16 $$\begin{aligned}&\frac{1}{N} \left( \left( \varvec{E}_K\right) ^T\varvec{E}_K\right) \mathop {\rightarrow }\limits ^{\tilde{\mathbb {P}}} C_{{{\textit{RP}}}} \text{ and } \nonumber \\&\frac{1}{\sqrt{N}} \sum _{i=1}^N \mathbf {e}_K(A_T(Z_i)) a_{T}^K(A_T(Z_i)) \mathop {\rightarrow }\limits ^{d} N\left( 0,\varvec{\Sigma }_{\text {RP}}\right) , \end{aligned}$$

where $N(0,\Sigma )$ denotes a normal distribution with mean 0 and covariance matrix $\Sigma$, $\mathop {\rightarrow }\limits ^{\tilde{\mathbb {P}}}$ denotes convergence in probability and $\mathop {\rightarrow }\limits ^{d}$ denotes convergence in distribution. Then, by the standard representation of the empirical error of least squares estimators and Slutsky’s lemma, it follows that

$$\begin{aligned}&\sqrt{N} ( {\hat{\varvec{\beta }}}_K- \varvec{\beta }_K ) \\&\quad =\sqrt{N}\left( \left( \varvec{V}_K\right) ^T\varvec{V}_K\right) ^{-1} \left( \varvec{V}_K\right) ^T (\varvec{a}_{0,t}^K+\varvec{p}_{0,t}) \mathop {\rightarrow }\limits ^{d} N\left( 0,(C_{{{\text {lsmc}}}})^{-1}\varvec{\Sigma }_{\text {lsmc}}(C_{{{\text {lsmc}}}})^{-1}\right) , \end{aligned}$$

where $$\varvec{a}_{0,t}^K=(a_{0,t}(A_t(Z_1)),\ldots ,a_{0,t}(A_t(Z_N)))^T$$ and

$\varvec{p}_{0,t}=(p_{0,t}(A_T(Z_1)),\ldots ,p_{0,t}(A_T(Z_N)))^T$. By the same argument

$$\begin{aligned}&\sqrt{N} \left( {\hat{\varvec{\alpha }}}_K- \varvec{\alpha }_K\right) \\&\quad =\sqrt{N}\left( \left( \varvec{E}_K\right) ^T\varvec{E}_K\right) ^{-1} \left( \varvec{E}_K \right) ^T \varvec{a}_T^K \mathop {\rightarrow }\limits ^{d} N\left( 0,(C_{{{\textit{RP}}}})^{-1}\varvec{\Sigma }_{\text {RP}}(C_{{{\textit{RP}}}})^{-1}\right) \end{aligned}$$

where $\varvec{a}_T^K=(a_T^K(A_T(Z_1)),\ldots ,a_T^K(A_T(Z_N)))^T$. Assume that the data $(X_i,A_t(Z_i))$, $i=1,\ldots ,N$, are i.i.d., then by the orthogonality of $g_{0,t}(A_t(Z))$ and $a_{0,t}^K(A_t(Z))+p_{0,t}(A_T(Z))$ , the second part of conditions (3.15) holds and (3.15) holds with $C_{{{\text {lsmc}}}}=I_K$, where $I_K$ denotes the $K \times K$ identity matrix, and

3.17 $$\begin{aligned} \varvec{\Sigma }_{\text {lsmc}} = \mathbb {E}_{\tilde{\mathbb {P}}} \left[ \left( a_{0,t}^K(A_t(Z))+p_{0,t}(A_T(Z))\right) ^2 \varvec{v}_K\left( A_t(Z)\right) \left( \varvec{v}_K\left( A_t(Z)\right) \right) ^T \right] . \end{aligned}$$

Similarly, if the data $(X_i,A_T(Z_i))$, $i=1,\ldots ,n$, are i.i.d., then (3.16) holds with $C_{{{\textit{RP}}}}=I$ and

3.18 $$\begin{aligned} \varvec{\Sigma }_{\text {RP}} = \mathbb {E}_{\tilde{\mathbb {P}}} \left[ \left( a_{T}^K(A_T(Z))\right) ^2 \varvec{e}_K\left( A_T(Z)\right) \left( \varvec{e}_K \left( A_T(Z)\right) \right) ^T \right] . \end{aligned}$$

The two asymptotic covariance matrices (3.17) and (3.18) in the i.i.d. case basically differ by the terms $a_{0,t}^K(A_t(Z))+p_{0,t}(A_T(Z))$ and $a_{T}^K(A_T(Z))$, because

$$\begin{aligned} \mathbb {E}_{\tilde{\mathbb {P}}} \left[ \varvec{v}_K \left( A_t(Z)\right) \left( \varvec{v}_K \left( A_t(Z)\right) \right) ^T \right] =\mathbb {E}_{\tilde{\mathbb {P}}} \left[ \varvec{e}_K \left( A_T(Z)\right) \left( \varvec{e}_K \left( A_T(Z)\right) \right) ^T \right] =I_K. \end{aligned}$$

We stress that in principle the functions $g_T$ and $g_{0,t}$ are quite different in various aspects, for example, they may differ in their dimensionality, so that a general comparison of $\varvec{\Sigma }_{\text {lsmc}}$ and $\varvec{\Sigma }_{\text {RP}}$ may not be feasible. We will come back to the potential differences in the structures of $g_T$ and $g_{0,t}$ in Sect. 4. However, if $g_T$ and $g_{0,t}$ have a similar structure so that $a_{0,t}^K$ and $a_T^K$ also have a similar structure, we expect the asymptotic covariance matrix of the LSMC estimator to be larger than the asymptotic covariance matrix of the replicating portfolio estimator due to the projection term $p_{0,t}$ in the LSMC asymptotic covariance matrix. Hence, then it should hold that

3.19 $$\begin{aligned} \varvec{\Sigma }_{\text {RP}} \le \varvec{\Sigma }_{\text {lsmc}} \end{aligned}$$

meaning, by Loewner’s ordering,

3.20 $$\begin{aligned} \varvec{\Sigma }_{\text {diff}} = \varvec{\Sigma }_{\text {lsmc}}-\varvec{\Sigma }_{\text {RP}} \end{aligned}$$

is a positive semidefinite matrix [see Definition 1.1, [38]]. Thus, if the approximation errors in LSMC and portfolio replication have a similar structure, then we can expect the variance of the replicating portfolio estimator to be smaller than the variance of the LSMC estimator, meaning that with portfolio replication we can yield a more accurate estimate. We next empirically analyze the property using the same basis for LSMC and portfolio replication given a function where the payoff and the conditional expectation function are similar in their structure.

#### Example 9

(Exponential with indicator functions)

Let the target variable *X* be the payoff from a geometric Brownian motion at time *T* on a compact domain,

3.21 $$\begin{aligned} X = e^{-\frac{1}{2} \sigma ^2 T + \sigma W(T)}; \quad W(T) \in [-2 \sqrt{T}, 2 \sqrt{T}]. \end{aligned}$$

We construct an orthornormal basis on $L_2(\mathbb {R}, \mathcal {B}(\mathbb {R}), \mathbb {P})$ based on nonoverlapping indicator functions. Consider the stochastic risk factor *W*(*T*) with probability measure $\mathbb {P}$. The domain $\mathbb {R}$ is chopped into *K* intervals, $\lbrace [b_1,b_2), [b_2,b_3), \ldots , [b_K, b_{K+1}) \rbrace$, such that ${\textit{Pr}}\left( b_k \le W(T) < b_{k+1}\right) = 1 \slash K$, $\forall k=1,\ldots ,K$. Defining *K* nonoverlapping indicator functions

3.22 $$\begin{aligned} \mathbbm {1}_{k}(W(T)) := {\left\{ \begin{array}{ll} 1 &{} \quad \text{ if } W(T) \in [b_{k}, b_{k+1}) \\ 0 &{} \quad \text {otherwise} \end{array}\right. } \end{aligned}$$

for $k=1,\ldots , K$. By construction, the indicator functions are orthogonal. Hence,

3.23 $$\begin{aligned} \mathbb {E}_{\mathbb {P}}\left[ \mathbbm {1}_j(W(T)) \mathbbm {1}_l(W(T))\right] = {\left\{ \begin{array}{ll} \frac{1}{K} &{}\quad \text {if } j=l \\ 0 &{} \quad \text {otherwise}. \end{array}\right. } \end{aligned}$$

Note that the set of indicator functions $\lbrace \sqrt{K} \; \mathbbm {1}_k(W_T) \rbrace _{k=1}^{\infty }$ is a basis for the Hilbert space [see Theorem 7.8, [25]]. The approximation to $X=g_T(W(T))$ is then

3.24 $$\begin{aligned} g_T^K(W(T)) = \sqrt{K} \sum _{k=1}^K \alpha _k \mathbbm {1}_k(W(T)) \end{aligned}$$

with

3.25 $$\begin{aligned} \alpha _k = \sqrt{K} \left( \Phi \left( \frac{b_{k+1}}{\sqrt{T}}-\sigma \sqrt{T}\right) - \Phi \left( \frac{b_k}{\sqrt{T}}-\sigma \sqrt{T}\right) \right) , \end{aligned}$$

where $\Phi (\cdot )$ denotes the cumulative standard normal distribution function. From Equation (3.18), the expectations are estimated based on simulations of $W_T$ with sample size 1,000,000 and parameters $\sigma =0.2$, $T=10$ and $t=1$.

In LSMC, the target variable to be replicated is the conditional expectation function $g_{0,t}(W(t))$,

3.26 $$\begin{aligned} g_{0,t}(W(t)) = e^{-\frac{1}{2} \sigma ^2 t + \sigma W(t)}; \quad W(t) \in [-2 \sqrt{t}, 2 \sqrt{t}]. \end{aligned}$$

Analogously to the portfolio replication case, we construct a basis of indicator functions for the LSMC problem. The domain $\mathbb {R}$ is chopped into *K* intervals, $\lbrace [a_1,a_2), [a_2,a_3),\ldots , [a_K, a_{K+1}) \rbrace$, such that ${\textit{Pr}}\left( a_k \le W(t) < a_{k+1}\right) = 1 \slash K$, $\forall k=1,\quad ,K$. Defining *K* nonoverlapping indicator functions

3.27 $$\begin{aligned} \mathbbm {1}_{k}(W(t)) := {\left\{ \begin{array}{ll} 1 &{} \quad \text{ if } W(t) \in [a_{k}, a_{k+1}) \\ 0 &{} \quad \text {otherwise} \end{array}\right. } \end{aligned}$$

for $k=1,\quad , K$. By construction, the indicator functions are orthogonal. The approximation to $g_{0,t}(W(t))$ is then

3.28 $$\begin{aligned} g_{0,t}^K(W(t)) = \sqrt{K} \sum _{k=1}^K \beta _k \mathbbm {1}_k(W(t)) \end{aligned}$$

with

3.29 $$\begin{aligned} \beta _k = \sqrt{K} \left( \Phi \left( \frac{a_{k+1}}{\sqrt{t}}-\sigma \sqrt{t}\right) - \Phi \left( \frac{a_k}{\sqrt{t}}-\sigma \sqrt{t}\right) \right) . \end{aligned}$$

The entries of the LSMC asymptotic covariance matrix in Eq. (3.17) are estimated based on simulating 1,000,000 sample paths of the standard Brownian motion from time *t* to *T*. Table 2 gives the eigenvalues of $\varvec{\Sigma }_{\text {lsmc}}-\varvec{\Sigma }_{\text {RP}}$ for $K=2,5,10$. The eigenvalues for $K=50,70$ have also been calculated, but to save space are not included in the table. The results indicate in every case that $\varvec{\Sigma }_{\text {diff}}$ is positive semidefinite.

**Table 2:** Eigenvalues of $\Sigma _{{\text {lsmc}}}-\Sigma _{{\textit{RP}}}$ for different *K*

	$K = 2$	$K=5$	$K=10$
Eigenvalues	0.12374054	0.13279645	0.11529827
	0.09337982	0.12104551	0.11289384
		0.11074061	0.10271642
		0.09129928	0.09111993
		0.06819562	0.08367246
			0.07561172
			0.06906777
			0.06209503
			0.05424688
			0.04384077

### Asymptotic measure independence in portfolio replication

In both LSMC and portfolio replication, we are searching for the coefficients of the basis terms that make up their respective representations. Looking at Eqs. (2.2) and (2.10), the coefficients depend on the measure $\tilde{\mathbb {P}}$. In many cases, it may be desirable to calibrate the representation under a different measure. For example, in order to sufficiently capture the tails of the target function, we may want to simulate more tail values of the underlying risk factors. Changing the measure, however, affects the result for the coefficients, meaning that we may not obtain the correct representation of the target function given a basis. In this section, we show that the replicating portfolio method is asymptotically measure-independent, but the LSMC result always depends on the chosen calibration measure. We will again see that the cause of this difference between LSMC and portfolio replication is linked to the nonzero projection error in LSMC.

Let us first discuss the portfolio replication case. Let $\varvec{E}$ be the orthonormal basis under $\tilde{\mathbb {P}}$. Let $\tilde{\mathbb {Q}}$ be a measure equivalent to $\tilde{\mathbb {P}}$. We first assume that we can perfectly replicate the target payoff function *X*, meaning that

$$\begin{aligned} \varvec{X} = \varvec{E} \varvec{\alpha }. \end{aligned}$$

We want to investigate whether the coefficients $\varvec{\alpha }$ can be found under both $\tilde{\mathbb {P}}$ and $\tilde{\mathbb {Q}}$. Let us first calculate the coefficients under $\tilde{\mathbb {P}}$

$$\begin{aligned} \varvec{\alpha }_{\tilde{\mathbb {P}}}&= \mathbb {E}_{\tilde{\mathbb {P}}}\left[ \varvec{E}^T \varvec{X}\right] \\&= \mathbb {E}_{\tilde{\mathbb {P}}}\left[ \varvec{E}^T \varvec{E} \varvec{\alpha } \right] \\&= \mathbb {E}_{\tilde{\mathbb {P}}}\left[ \varvec{E}^T \varvec{E} \right] \varvec{\alpha } \\&= \varvec{\alpha } \end{aligned}$$

since $\mathbb {E}_{\tilde{\mathbb {P}}}\left[ \varvec{E}^T \varvec{E}\right] = \varvec{I}$ due to the orthonormality of the basis under $\tilde{\mathbb {P}}$, where $\varvec{I}$ is the identity matrix. Now, when we change the measure to $\tilde{\mathbb {Q}}$ , the basis may not still be orthonormal. Hence, the coefficients are calculated as

$$\begin{aligned} \varvec{\alpha }_{\tilde{\mathbb {Q}}}&= \left( \mathbb {E}_{\tilde{\mathbb {Q}}}\left[ \varvec{E}^T \varvec{E} \right] \right) ^{-1} \mathbb {E}_{\tilde{\mathbb {Q}}}\left[ \varvec{E}^T \varvec{X} \right] \\&= \left( \mathbb {E}_{\tilde{\mathbb {Q}}}\left[ \varvec{E}^T \varvec{E} \right] \right) ^{-1} \mathbb {E}_{\tilde{\mathbb {Q}}}\left[ \varvec{E}^T \varvec{E} \varvec{\alpha } \right] \\&= \left( \mathbb {E}_{\tilde{\mathbb {Q}}}\left[ \varvec{E}^T \varvec{E} \right] \right) ^{-1} \mathbb {E}_{\tilde{\mathbb {Q}}}\left[ \varvec{E}^T \varvec{E} \right] \varvec{\alpha } \\&= \varvec{\alpha }. \end{aligned}$$

Thus, when the perfect basis is available, the correct coefficients are obtained independent of the measure. Note that this does actually not depend on the orthonormality property of the basis, i.e. it also holds when $\varvec{E}$ is not orthonormal under either $\tilde{\mathbb {P}}$ or $\tilde{\mathbb {Q}}$. Now let us consider the portfolio replication case, where we have an approximation error, i.e.

$$\begin{aligned} \varvec{X} = \varvec{E}^K \varvec{\alpha }^K + \varvec{a}_T^K, \end{aligned}$$

where $\varvec{E}^K$ contains the truncated basis, i.e. *K* basis terms, and $\varvec{\alpha }^K$ denotes the *K* true coefficients of the truncated basis terms, and $\varvec{a}_T^K$ denotes the approximation error. We are looking for the coefficients $\varvec{\alpha }^K$, which we again correctly obtain under $\tilde{\mathbb {P}}$,

$$\begin{aligned} \varvec{\alpha }_{\tilde{\mathbb {P}}}^K&= \mathbb {E}_{\tilde{\mathbb {P}}}\left[ (\varvec{E}^K)^T \varvec{X}\right] \\&= \mathbb {E}_{\tilde{\mathbb {P}}}\left[ (\varvec{E}^K)^T (\varvec{E}^K \varvec{\alpha }^K + \varvec{a}_T^K)\right] \\&= \mathbb {E}_{\tilde{\mathbb {P}}}\left[ (\varvec{E}^K)^T \varvec{E}^K \varvec{\alpha }^K \right] + \mathbb {E}_{\tilde{\mathbb {P}}}\left[ (\varvec{E}^K)^T \varvec{a}_T^K \right] \\&= \varvec{\alpha }^K, \end{aligned}$$

since by the orthonormality of the basis $\varvec{E}^K$ and $\varvec{a}_T^K$ are orthogonal and

$\mathbb {E}_{\tilde{\mathbb {P}}}\left[ (\varvec{E}^K)^T \varvec{E}^K \right] = \varvec{I}$. Changing the measure to $\tilde{\mathbb {Q}}$ yields

$$\begin{aligned} \varvec{\alpha }_{\tilde{\mathbb {Q}}}^K&= \left( \mathbb {E}_{\tilde{\mathbb {Q}}}\left[ (\varvec{E}^K)^T \varvec{E}^K \right] \right) ^{-1}\mathbb {E}_{\tilde{\mathbb {Q}}}\left[ (\varvec{E}^K)^T \varvec{X}\right] \\&= \left( \mathbb {E}_{\tilde{\mathbb {Q}}}\left[ (\varvec{E}^K)^T \varvec{E}^K \right] \right) ^{-1} \left( \mathbb {E}_{\tilde{\mathbb {Q}}}\left[ (\varvec{E}^K)^T \varvec{E}^K \right] \varvec{\alpha }^K + \mathbb {E}_{\tilde{\mathbb {Q}}}\left[ (\varvec{E}^K)^T \varvec{a}_T^K \right] \right) \\&= \varvec{\alpha }^K + \left( \mathbb {E}_{\tilde{\mathbb {Q}}}\left[ (\varvec{E}^K)^T \varvec{E}^K \right] \right) ^{-1}\mathbb {E}_{\tilde{\mathbb {Q}}}\left[ (\varvec{E}^K)^T \varvec{a}_T^K \right] . \end{aligned}$$

We see that when there is an approximation error changing the measure does not yield the correct coefficients. Only when $K \rightarrow \infty$, the approximation error converges to zero and $\varvec{\alpha }_{\tilde{\mathbb {Q}}}^K \rightarrow \varvec{\alpha }^K$. Hence, asymptotically, the replicating portfolio technique is measure-independent.

Let us now investigate the LSMC case. We denote the basis at time *t* by $\varvec{V}$, which is orthonormal under $\tilde{\mathbb {P}}$. Then, we can write

$$\begin{aligned} \varvec{X} = \varvec{V}^K \varvec{\beta }^K + \varvec{a}_{0,t}^K + \varvec{p}_{0,t}, \end{aligned}$$

where $\varvec{V}^K$ denotes the truncated basis, $\varvec{a}_{0,t}^K$ denotes the approximation error and $\varvec{p}_{0,t}$ is the projection error. We again first calculate the coefficients under the measure $\tilde{\mathbb {P}}$.

$$\begin{aligned} \varvec{\beta }_{\tilde{\mathbb {P}}}^K&= \mathbb {E}_{\tilde{\mathbb {P}}}\left[ (\varvec{V}^K)^T \varvec{X}\right] \\&= \mathbb {E}_{\tilde{\mathbb {P}}}\left[ (\varvec{V}^K)^T (\varvec{V}^K \varvec{\beta }^K + \varvec{a}_{0,t}^K + \varvec{p}_{0,t})\right] \\&= \varvec{\beta }^K + \mathbb {E}_{\tilde{\mathbb {P}}}\left[ (\varvec{V}^K)^T \varvec{a}_{0,t}^K \right] + \mathbb {E}_{\tilde{\mathbb {P}}}\left[ (\varvec{V}^K)^T \varvec{p}_{0,t} \right] \\&= \varvec{\beta }^K \end{aligned}$$

since the approximation error and the basis terms up to *K* are orthogonal by construction and the projection error is orthogonal to each basis term at time *t*. Changing the measure to $\tilde{\mathbb {Q}}$ gives

$$\begin{aligned} \varvec{\beta }_{\tilde{\mathbb {Q}}}^K&= \left( \mathbb {E}_{\tilde{\mathbb {Q}}}\left[ (\varvec{V}^K)^T \varvec{V}^K \right] \right) ^{-1}\mathbb {E}_{\tilde{\mathbb {Q}}}\left[ (\varvec{V}^K)^T \varvec{X} \right] \\&= \left( \mathbb {E}_{\tilde{\mathbb {Q}}} \left[ (\varvec{V}^K)^T \varvec{V}^K \right] \right) ^{-1} \mathbb {E}_{\tilde{\mathbb {Q}}}\left[ (\varvec{V}^K)^T (\varvec{V}^K \varvec{\beta }^K + \varvec{a}_{0,t}^K + \varvec{p}_{0,t}) \right] \\&= \varvec{\beta }^K + \left( \mathbb {E}_{\tilde{\mathbb {Q}}}\left[ (\varvec{V}^K)^T \varvec{V}^K \right] \right) ^{-1} \left( \mathbb {E}_{\tilde{\mathbb {Q}}}\left[ (\varvec{V}^K)^T \varvec{a}_{0,t}^K \right] + \mathbb {E}_{\tilde{\mathbb {Q}}}\left[ (\varvec{V}^K)^T \varvec{p}_{0,t} \right] \right) . \end{aligned}$$

Even if the approximation error is zero, i.e. $K \rightarrow \infty$ we have

$$\begin{aligned} \varvec{\beta }_{\tilde{\mathbb {Q}}} = \varvec{\beta } + \left( \mathbb {E}_{\tilde{\mathbb {Q}}}\left[ (\varvec{V}^K)^T \varvec{V}^K \right] \right) ^{-1}\mathbb {E}_{\tilde{\mathbb {Q}}}\left[ (\varvec{V}^K)^T \varvec{p}_{0,t} \right] . \end{aligned}$$

Thus, even in the limit when the approximation error is zero, the projection error remains and changing the measure affects the coefficients obtained.

Summing up, as the approximation error vanishes, the replicating portfolio constructed with the least squares Monte Carlo method of Sect. 2.2 is perfect regardless of the measure used for calibration. For LSMC, the situation is different. Even if the approximation error is zero, the projection error is nonzero, since in LSMC $t < T$. Consequently, even in the limit, the LSMC estimator is measure-dependent. We will illustrate this result for LSMC and portfolio replication with several simple examples. Note that we refrain from orthonormalizing the basis as the examples are more intuitive using the nonorthonormalized basis. Nonetheless, we could, of course, orthonormalize these basis terms to be consistent with the presented theory. Moreover, it can easily be shown that the conclusions made above on the measure dependence also hold if a nonorthonormal basis is used.

#### Example 10

(Simple Brownian motion)

First, we again use the very simple Brownian motion case of Example 1, in which both for LSMC and portfolio replication the approximation errors are equal to zero. Recall that for this example we have $X = W_T$ and $\mathbb {E}[X | \mathcal {F}_t] = W_t$. For the LSMC approach, the basis is $W_t$ , while for the portfolio replication approach, the basis is $W_T$. We write down the following regression equations with constants

$$\begin{aligned}&W_T = \alpha _0 + \alpha _1 W_T \\&W_T = \beta _0 + \beta _1 W_t + p_{0,t}(W_T) \end{aligned}$$

The correct coefficients are $\alpha _0, \beta _0=0$ and $\alpha _1, \beta _1 = 1$.

**Fig. 17:**
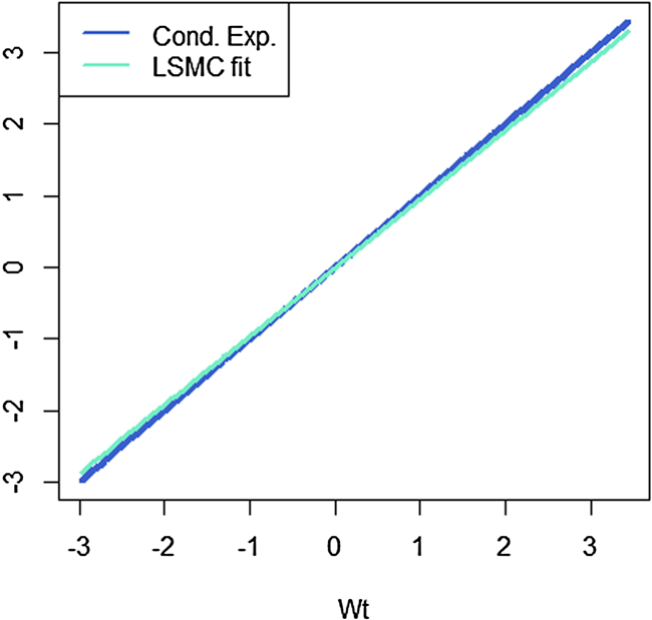
LSMC fit with calibration on correct measure (Example 10)

**Fig. 18:**
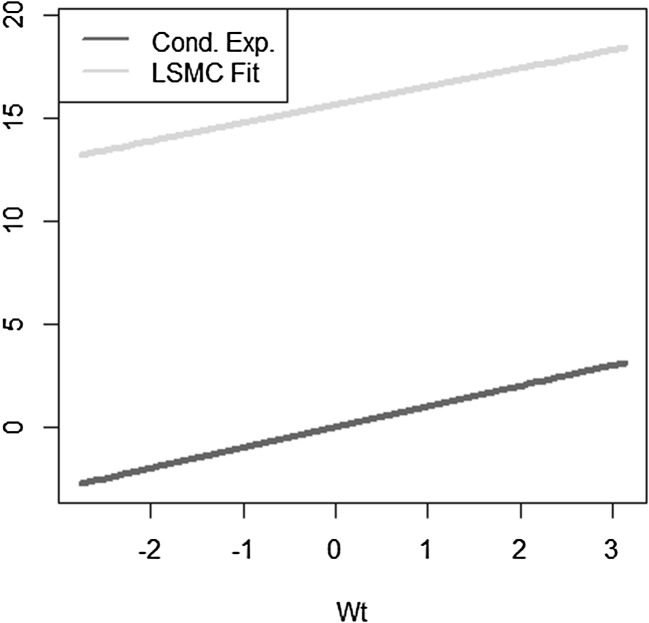
LSMC fit with calibration on shifted normal (Example 10)

Instead of simulating the Brownian motions from the normal distribution, we run the least squares regression based on a sample of size 1000 simulated from the shifted normal distribution with mean $\mu = 5$. Hence, we simulate $W_t=\sqrt{t} Z$ and $W_T = W_t + \sqrt{T-t} Z$ with $Z \sim N(\mu ,1)$. For the portfolio replication approach, the change of measure has no effect since the perfect replicating portfolio is still simply the Brownian motion at time *T*, i.e. $W_T$. Thus, we obtain the correct coefficients $\alpha _0 = 0$ and $\alpha _1 = 1$. However, the LSMC estimate gives $\hat{\beta }_0 = 15.65$ and $\hat{\beta }_1 = 0.88$. The coefficients make sense considering that the conditional expectation function under the shifted normal measure is now $g_t(W_t) = \sqrt{T-t} \, \mu + W_t$. After all, however, the goal is to achieve a fit to the conditional expectation function under the original measure. Clearly, in LSMC, we cannot easily switch to a different measure for calibrating the fitting function. We evaluate the out-of-sample fit of both regression approaches based on a sample that has not been used for calibration. As for portfolio replication, the correct coefficients were obtained and the out-of-sample fit is perfect. However, for LSMC, the coefficients are biased due to the calibration based on the shifted normal distribution. Figure 17 gives the LSMC result calibrated based on the normal distribution. Figure 18 shows the fit for the LSMC estimation calibrated based on the shifted normal distribution.

#### Example 11

(Exponential function)

Let us take Example 2, but consider *Z* in $W_t = \sqrt{t} Z$ and $W_T = \sqrt{T} Z$ to be simulated from a truncated normal on $[-2,2]$. We compare the goodness of fit for both LSMC and portfolio replication when calibrating under the truncated normal distribution and when calibrating under the uniform on $[-2,2]$. Note that for the case at hand an approximation error is present in both LSMC and portfolio replication.

**Fig. 19:**
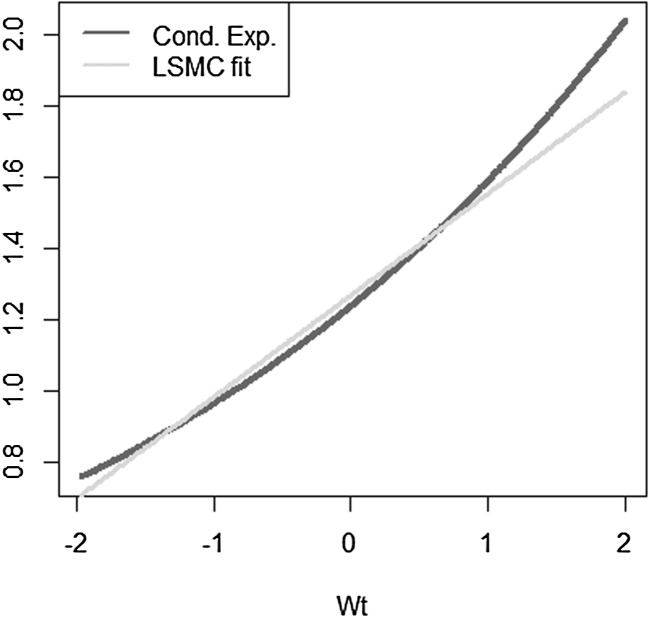
LSMC fit with calibration on correct measure (Example 11)

**Fig. 20:**
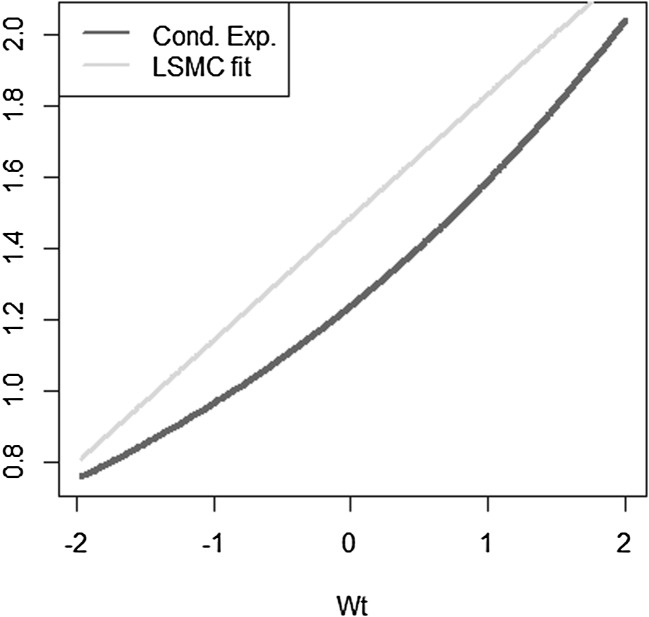
LSMC fit with calibration on uniform (Example 11)

**Fig. 21:**
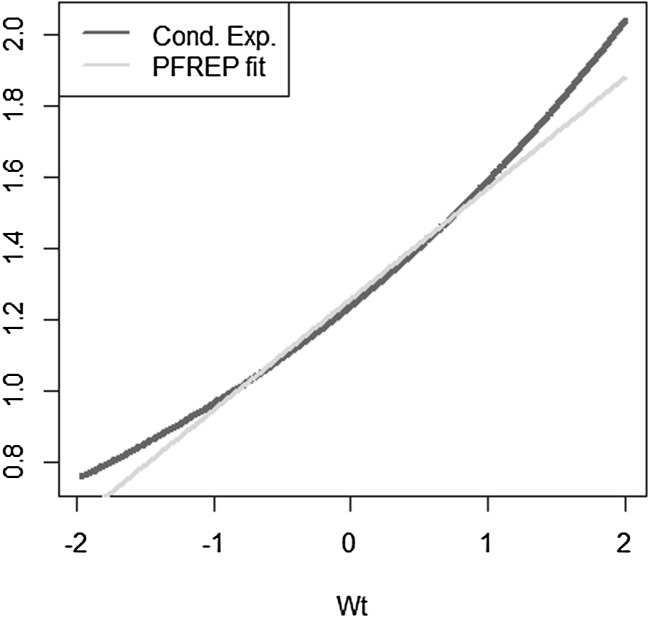
Replication fit with calibration on correct measure (Example 11)

**Fig. 22:**
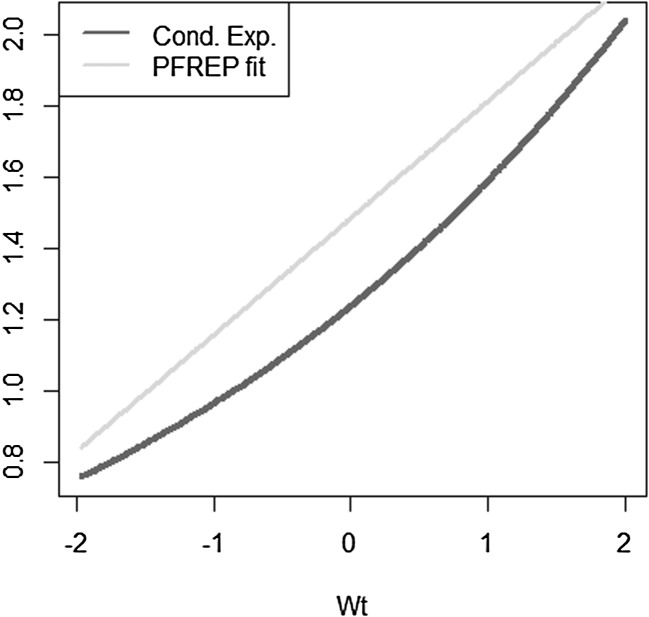
Replication fit with calibration on uniform (Example 11)

Figures 19 and 20 compare the LSMC out-of-sample goodness of fit for the calibration on the true measure and on the uniform. Figures 21 and 22 are the analog for portfolio replication. We clearly see that for the example at hand both LSMC and portfolio replication are measure-dependent, meaning that the coefficient estimates depend on the measure we use for calibration. While we have already seen in the previous example that LSMC is measure-dependent, the measure dependence here for the replicating portfolio results from the approximation error.

#### Example 12

(Artificial portfolio)

In this example, we construct a payoff function from a set of calls and puts. We define the target payoff function as

$$\begin{aligned} X&= 100- 2(K_1-S(T))^{+} + (S(T)-K_2)^{+} - 2 (S(T)-K_3)^{+} + (S(T)-K_4)^{+} \\&\quad + 0.5 (S(T)-K_5)^{+} -0.5 (S(T)-K_6)^{+} \end{aligned}$$

with $K_1$ to $K_6$ given by $\lbrace 20,50,100,150,200,205 \rbrace$.

**Fig. 23:**
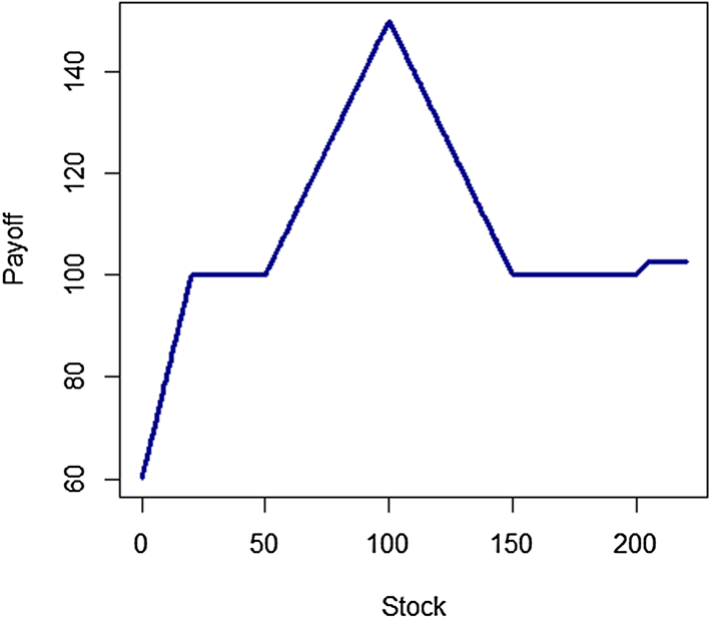
Payoff function of *X* (Example 12)

Note that we have deliberately constructed the target function such that it changes in the tails at, for example, $S_T=20$. This is also the reason why the strikes in this example differ from Example 3. We plot the payoff function for *X* in Fig. 23. The underlying stock, denoted by *S*(*T*) at time *T*, is modeled as a geometric Brownian motion

3.30 $$\begin{aligned} S(T)&= S_0 \exp \left( \left( \mu -\frac{1}{2} \sigma ^2 \right) T + \sigma W(T)\right) \nonumber \\&= S(t) \exp \left( \left( \mu -\frac{1}{2} \sigma ^2 \right) (T-t) + \sigma (W(T)-W(t) \right) , \end{aligned}$$

where $W(\cdot )$ is a standard Brownian motion. The parameters are given in Table 1. We assume the Black–Scholes model and thus obtain the conditional expectation function $g_t(S(t))$ by pricing the calls and puts in *X* using the Black–Scholes formula. Note that we omit the subscript “0” in $g_t$ as the conditional expectation function is known for the case at hand. Ultimately, with LSMC and portfolio replication, we want to obtain an approximation of the Black–Scholes price of *X*.

As basis, we choose eight terms consisting of a constant (zero-coupon bond), the underlying stock and a series of puts on the underlying stock with strikes $\lbrace 18,48,98,148,198,203 \rbrace$. We want to investigate the measure dependence for both LSMC and portfolio replication using different measures for calibration. We consider five different calibration scenario sets, each of size *N*, which contain the paths for $\lbrace S(t), S(T) \rbrace$, based on the real-world probability measure $\mathbb {P}$, the risk-neutral measure $\mathbb {Q}$ and the uniform measure. While for the calibration of the replicating portfolio we only need the values *S*(*T*), for the calibration of the LSMC representation we require both.

Under $\mathbb {P}$ the stock *S*(*T*) is modeled as in Eq. (3.30). Changing to the equivalent measure $\mathbb {Q}$ *S*(*T*) is modeled as

3.31 $$\begin{aligned} S(s)&= S_0 \exp \left( \left( r-\frac{1}{2} \sigma ^2 \right) T + \sigma W(T)\right) \nonumber \\&= S(t) \exp \left( \left( r-\frac{1}{2} \sigma ^2 \right) (T-t) + \sigma (W(T)-W(t)) \right) , \end{aligned}$$

where *r* is the risk-free rate. Under the uniform we simply simulate the stock values from the uniform. The sets are specified in Table 3. For sets one to four, the same random numbers for $W(T)-W(t) = \sqrt{T-t} \, Z$, $Z \sim N(0,1)$ are used to ensure that the difference in the sample truly comes from the difference between the measures $\mathbb {P}$ and $\mathbb {Q}$. Set five is constructed such that the range on which the target function *X* varies the most is sufficiently captured. Note that with set five the assumption on measure equivalence is violated as the measure in set five has a different domain than $\mathbb {P}$ and $\mathbb {Q}$. Even when violating this assumption, we will see that set five is helpful for our testing purposes. We assess the quality of fit based on an *m*-sized sample of paths for *S*(*t*) and *S*(*T*) that is sufficiently diverse to capture the range of values, on which *X* and $g_t(S(t))$ vary the most.

**Table 3:** Calibration sets for 12

Set 1: $\mathbb {P}$	*N* values generated from (3.30) with $S_0 = 100$
Set 2: $\mathbb {Q}$	*N* values generated from (3.31) with $S_0 = 100$
Set 3: $\mathbb {P}$ mixed	$N-400$ values generated from (3.30) with $S_0 = 100$ plus 200 values per $S_0=20$ and $S_0= 150$
Set 4: $\mathbb {Q}$ mixed	$N-400$ values generated from (3.31) with $S_0 = 100$ plus 200 values per $S_0=20$ and $S_0= 150$
Set 5: uniform	*S*(*t*) [0, 250], $S(T)=S(t)*[0,1.5]$

As an almost perfect basis is used for both the construction of the LSMC representation and the replicating portfolio the approximation error is small. Therefore, we expect the replicating portfolio approach to be rather measure-independent. As in LSMC, a projection error is additionally present and we expect to see measure-dependence when calibrating the LSMC representation under different measures.

Table 4 summarizes the results for the out-of-sample MSE and $R^2$ for both LSMC and portfolio replication. The out-of-sample $R^2$ is here calculated as the $R^2$ from regressing the fitted function $\hat{g}_t^K$ from LSMC and portfolio replication against the true function $g_t$. Note that for portfolio replication, we additionally provide the measures for the goodness of fit to the payoff function *X*.

**Table 4:** Results for Example 12

		Fit to *X*		Fit to $g_t(S(t))$	
		MSE	$R^2$ ^a^	MSE	$R^2$ ^b^
RP	Set 1	58.49935	0.8203814	57.9955	0.6385629
	Set 2	18.26597	0.9439155	16.60685	0.9119326
	Set 3	1.473218	0.9954766	0.1499509	0.9992251
	Set 4	1.480922	0.9954529	0.09592363	0.9993492
	Set 5	1.905271	0.99415	0.2598456	0.9983761
LSMC	Set 1			3.73E+18	0.2276543
	Set 2			5.55E+16	0.2276543
	Set 3			9.621861	0.9645457
	Set 4			0.5070824	0.9964742
	Set 5			250.848	0.6685076

$^{a}$For consistency (see the following footnote) calculated as the $R^2$ from regressing the fitted function $\hat{g}_T^K$ against the true function $g_T$.

$^{b}$Calculated as the $R^2$ from regressing the fitted function $\hat{g}_t^K$ from LSMC and portfolio replication against the true function $g_t$. For portfolio replication, the best results are attained when the calibration set is sufficiently diverse to capture the full range, on which the target function varies the most. Therefore, a comparably good fit is achieved under scenario sets three to five. The resulting out-of-sample fit when using calibration set five, for example, is illustrated in Fig. 25

**Fig. 24:**
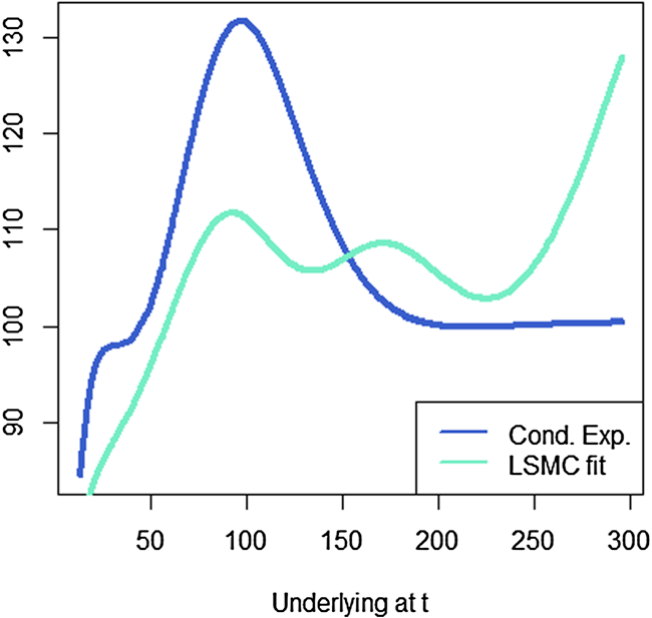
LSMC fit with calibration on uniform (Example 12)

**Fig. 25:**
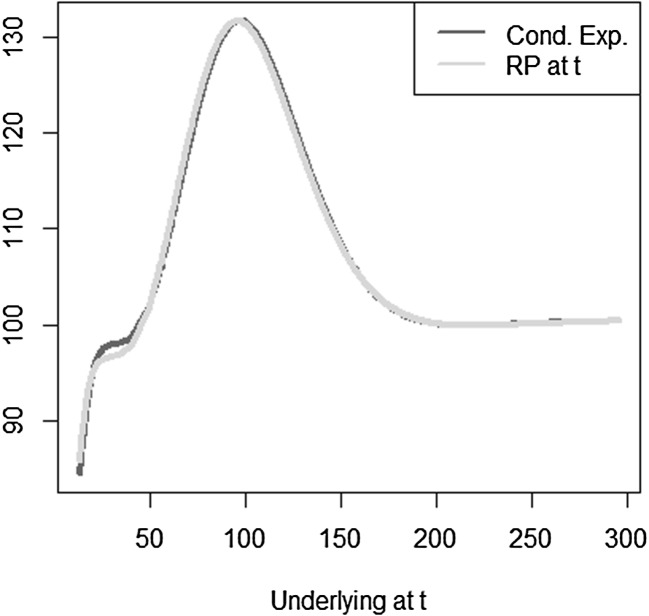
Replication fit with calibration on uniform (Example 12)

**Fig. 26:**
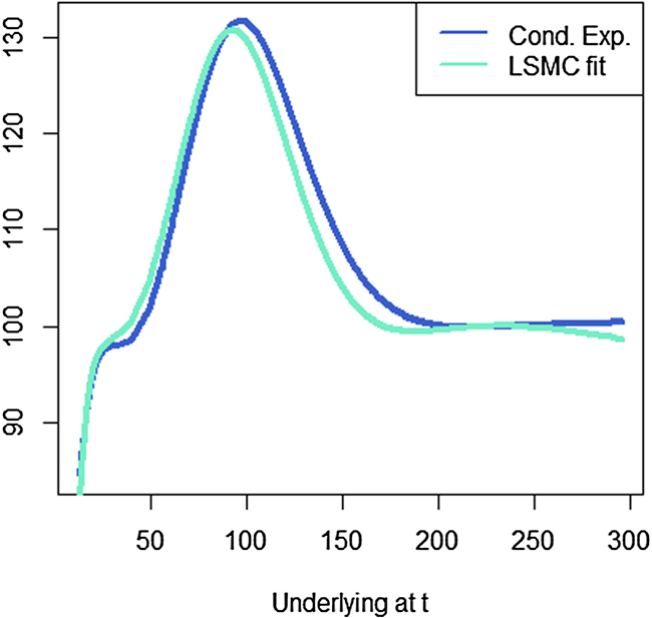
LSMC fit with calibration on set three (Example 12)

**Fig. 27:**
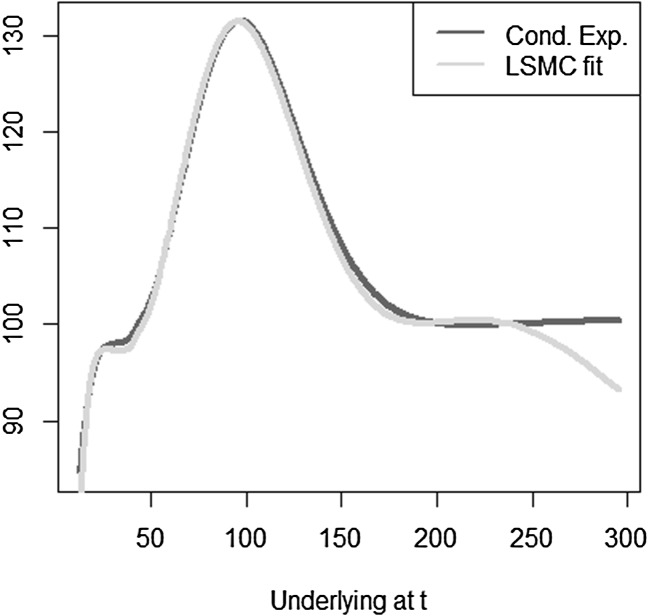
LSMC fit with calibration on set four (Example 12)

**Fig. 28:**
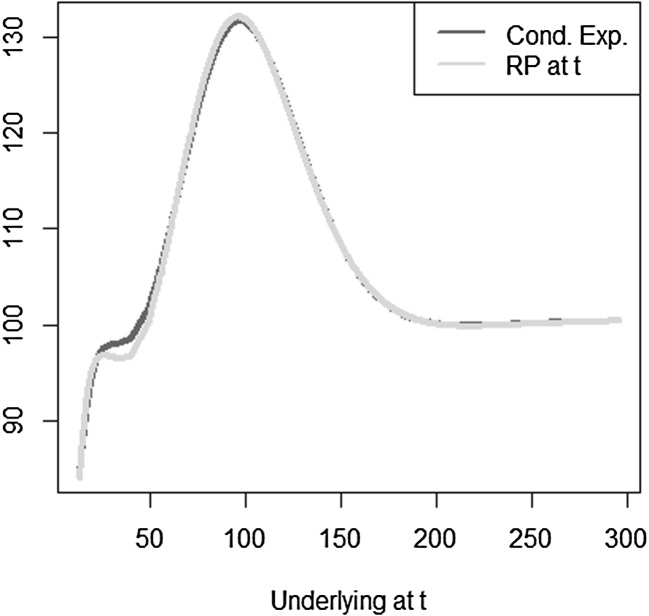
Replication fit with calibration on set three (Example 12)

**Fig. 29:**
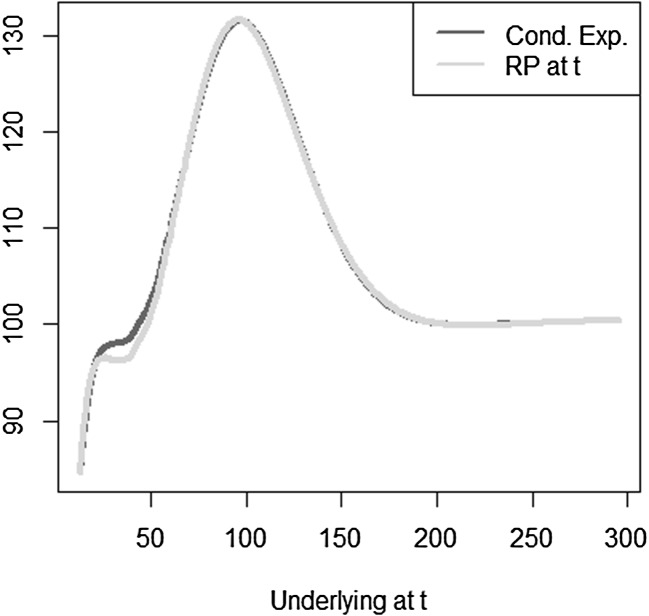
Replication fit with calibration on set four (Example 12)

In LSMC, the solution is measure-dependent. Clearly, calibrating under set five leads to a bad result (see Fig. 24). Nonetheless, a diverse scenario set is required in order to capture the tail behavior of the target function. Set four works best for the LSMC calibration. It is based on the risk-neutral measure, with which the conditional expectation function is calculated, and contains shock scenarios, which makes it more diverse compared to set two. Sets three and four both perform better than set two although set three is based on measure $\mathbb {P}$. This is due to the fact that set three is much more diverse than set two and this information is needed to calibrate the function properly in the tails. However, set four clearly outperforms set three in LSMC, while in the replicating portfolio approach sets three and four yield almost equal results. Sets three and four are very similar in their structure as the same random numbers have been used and the only difference is their growth rate $\mu$ and *r*. But the coefficients in LSMC are measure-dependent and we can see that from the resulting fits illustrated in Figs.26 and 27. For portfolio replication, almost the same portfolios are achieved with sets three and four (see Figs. 28 and 29).

We note that the last example is quite artificial in that we know exactly the range on which the target function varies and could construct the calibration and out-of-sample scenario sets accordingly. In practice, this information is mostly not available. In that case, we should decide for each risk factor on the range that is considered relevant. The calibration and out-of-sample scenario sets should then be created to sufficiently cover that range.

The following conclusions are drawn from the analysis and the examples.

- For LSMC use, a sufficiently diverse calibration set based on the measure under which the conditional expectation function is calculated.
- With portfolio replication, there is much more flexibility in choosing a measure when we expect the approximation error to be rather small. The recommendation is then to use a diverse set that sufficiently covers the relevant range. Calibrations using the uniform distribution have shown good results. The measure for calibrating the replicating portfolio may thus differ from the measure under which we want to find the conditional expectation function.

### Asymptotic convergence

In Sect. 2, we have given the mathematical model for LSMC and portfolio replication. We have seen that, given a basis on the relevant risk factors of the respective target functions *X* and $g_{0,t}$ , a perfect representation exists. However, the perfect representation may involve infinitely many basis terms, which complicates the problem of estimating the coefficients of the basis terms in finite samples. Therefore, the infinite representations are truncated to finite representations based on a finite number of *K* basis terms. In order to analyze the asymptotic convergence to the true result, both the truncation parameter *K* and the sample size *N* must grow simultaneously. In [7], the asymptotic convergence rate of LSMC regress-later, i.e. portfolio replication, is derived and compared to the asymptotic convergence rate of LSMC regress-now, i.e. LSMC in the terminology of this paper (see also [34, 39]). In this section, we briefly repeat the asymptotic convergence theorems and comment on the difference in the convergence rates for LSMC and portfolio replication. We refer to [7] for details and the proofs.

Let us first give the asymptotic convergence result for portfolio replication. Two assumptions are required.

#### Assumption 1

There are $$\gamma _{\textit{RP}} > 0$$, $$\varvec{\alpha }_K$$ s.t.

$$\begin{aligned}&\sqrt{ \mathbb {E}_{\tilde{\mathbb {P}}}\left[ \left( g_T(A_T(Z)) - (\varvec{\alpha }_K)^T \mathbf {e}_K(A_T(Z))\right) ^4\right] } \nonumber \\&\quad = \sqrt{\int _{\mathbb {R}^{\ell }} \left( g_T(u) - (\varvec{\alpha }_K)^T \mathbf {e}_K(u)\right) ^4 \, \mathrm {d}\tilde{\mathbb {P}}^{A_T(Z)}(u)} \nonumber \\&\quad = \sqrt{\int _{\mathbb {R}^{\ell }} a_T^K(u)^4 \, \mathrm {d}\tilde{\mathbb {P}}^{A_T(Z)}(u)} \nonumber \\&\quad =O\big (K^{- \gamma _{\textit{RP}}}\big ). \end{aligned}$$

Assumption 1 controls the convergence of the approximation error.

#### Assumption 2

$$\left( (X_{1}, A_T(Z_1)),\ldots ,(X_{N}, A_T(Z_{N}))\right) $$ are i.i.d.

Moreover, we define

$$\begin{aligned} \tilde{h}^{RP}(N,K):= \frac{1}{N} \; \mathbb {E}_{\tilde{\mathbb {P}}}\left[ \left( \left( \varvec{e}_K(A_T(Z))\right) ^T \varvec{e}_K(A_T(Z))\right) ^2\right] . \end{aligned}$$

Notice that $\tilde{h}^{{\textit{RP}}}(N,K)$ controls the growth rate of the truncation parameter *K* in relation to the sample size *N*. Intuitively, it is clear that such a growth rate is required in order to ensure that the sample size is sufficiently large to estimate a certain number of parameters. Now, we can give the theorem on the asymptotic convergence rate of the portfolio replication method.

#### Theorem 1

Let Assumptions 1 and 2 be satisfied. Additionally, assume that there is a sequence $\mathcal {K}: \mathbb {N}\rightarrow \mathbb {N}$ such that

3.32 $$\begin{aligned} \tilde{h}^{RP}(N,\mathcal {K}(N)) \rightarrow 0 \text{ as } N \rightarrow \infty . \end{aligned}$$

Then

$$\begin{aligned} \mathbb {E}_{\tilde{\mathbb {P}}}\left[ \left( X - \hat{g}_T^{\mathcal {K}(N)}(A_T(Z))\right) ^2 \right] = O_{\tilde{\mathbb {P}}}\left( \mathcal {K}(N)^{-\gamma _{RP}}\right) . \end{aligned}$$

#### Proof

See [7]. $$\square $$

Next, we present the asymptotic convergence theory for LSMC as stated in [7]. We again require two assumptions.

#### Assumption 3

There are $$\gamma _{lsmc} > 0$$, $$\varvec{\beta }_K$$ s.t.

$$\begin{aligned}&\sqrt{ \mathbb {E}_{\tilde{\mathbb {P}}}\left[ \left( g_{0,t}(A_t(Z)) - (\varvec{\beta }_K)^T \mathbf {v}_K(A_t(Z))\right) ^4\right] } \nonumber \\&\quad = \sqrt{\int _{\mathbb {R}^{s}} \left( g_{0,t}(u) - (\varvec{\beta }_K)^T \mathbf {v}_K(u)\right) ^4 \, \mathrm {d}\tilde{\mathbb {P}}^{A_t(Z)}(u)} \nonumber \\&\quad = \sqrt{\int _{\mathbb {R}^{s}} a_{0,t}^K(u)^4 \, \mathrm {d}\tilde{\mathbb {P}}^{A_t(Z)}(u)} \nonumber \\&\quad =O(K^{- \gamma _{lsmc}}). \end{aligned}$$

#### Assumption 4

$$\left( (X_{1}, A_t(Z_1)),\ldots ,(X_{N}, A_t(Z_{N}))\right) $$ are i.i.d. and

$$\mathbb {E}_{\tilde{\mathbb {P}}}\left[ \big (p_{0,t}(A_T (Z))\big )^2|A_t(Z)\right] =\sigma ^2$$.

Similarly as in portfolio replication, we also define

$$\begin{aligned} \tilde{h}^{lsmc}(N,K):= \frac{1}{N} \; \mathbb {E}_{\tilde{\mathbb {P}}}\left[ \left( \left( \varvec{v}_K(A_t(Z)) \right) ^T \varvec{v}_K(A_t(Z))\right) ^2\right] , \end{aligned}$$

which controls the growth rate of *K* in relation to *N*. We can now state the theorem.

#### Theorem 2

Let Assumptions 3 and 4 be satisfied. Additionally, assume that there is a sequence $\mathcal {K}: \mathbb {N} \rightarrow \mathbb {N}$ such that

3.33 $$\begin{aligned} \tilde{h}^{\text {lsmc}}(N,\mathcal {K}(N)) \rightarrow 0 \text{ as } N \rightarrow \infty . \end{aligned}$$

Then

3.34 $$\begin{aligned} \mathbb {E}_{\tilde{\mathbb {P}}}\left[ \left( g_{0,t}(A_t(Z)) - \hat{g}_{0,t}^{\mathcal {K}(N)}(A_t(Z))\right) ^2 \right] = O_{\tilde{\mathbb {P}}}\left( \frac{\mathcal {K}(N)}{N}+\mathcal {K}(N)^{-\gamma _{lsmc}}\right) . \end{aligned}$$

#### Proof

See [7]. $$\square $$

The difference in the convergence rate of LSMC and portfolio replication depends on $\gamma _{\textit{RP}}$ and $\gamma _{\text {lsmc}}$. Moreover, the LSMC convergence rate additionally contains the term $\mathcal {K}(N) \slash N$, which is not present in portfolio replication. In [7], it is shown that this additional term in LSMC is driven by its nonzero projection error. The absence of the term $\mathcal {K}(N) \slash N$ in the mean-square error of portfolio replication makes it plausible that the replicating portfolio estimator may potentially converge faster than the LSMC estimator. We deliberately state here “potentially” as the ultimate convergence rate depends on the $\gamma _{\text {lsmc}}$ and $\gamma _{\textit{RP}}$ which are problem-dependent. In particular, the choice of basis plays an important role in the determination of $\gamma _{\text {lsmc}}$ and $\gamma _{\textit{RP}}$. However, the LSMC convergence rate can never be faster than $N^{-1}$. This follows simply from the fact that the best we can hope for is that $g_{0,t}$ is contained in the span of finitely many basis functions. Then the approximation error vanishes and we are left with the rate $N^{-1}$. In contrast, in portfolio replication if Condition (3.32) is fulfilled with $\mathcal {K}(N) \propto N^a$ for some $0<a<1$, then the convergence rate for the replicating portfolio equals $N^{-a \; \gamma _{\textit{RP}}}$. We can see that for the right combination of *a* and $\gamma _{\textit{RP}}$ it is possible to achieve a convergence rate that is even faster than $N^{-1}$.

We want to remark on one further point. The discussed general convergence rates pertain to convergence to different functions. While in LSMC the convergence rate pertains to convergence to the unknown conditional expectation function $g_{0,t}(A_t(Z))$, the convergence rate for replicating portfolios pertains to convergence to the true payoff function *X*. Ultimately in the context of Solvency II insurers are interested in the time *t* value of its liabilities under different scenarios for the underlying risk drivers. While in LSMC we directly have this, in portfolio replication we achieve the approximation to the conditional expectation function by applying the conditional expectation operator to the estimated payoff function, $\hat{g}_T^{\mathcal {K}(N)}$. We can show that the ultimate estimator given by the conditional expectation of the estimator of *X* does not converge slower than at the rate derived for the convergence of $\hat{g}_T^K(A_T(Z))$. More explicitly we have

$$\begin{aligned}&\mathbb {E}_{\tilde{\mathbb {P}}} \left[ \left( \mathbb {E}_{\tilde{\mathbb {P}}}[X | \mathcal {F}_t]- \mathbb {E}_{\tilde{\mathbb {P}}}\left[ \hat{g}_T^{\mathcal {K}(N)}(A_T(Z)) \big | \mathcal {F}_t\right] \right) ^2\right] \\&\quad = \mathbb {E}_{\tilde{\mathbb {P}}}\left[ \left( \mathbb {E}_{\tilde{\mathbb {P}}}\left[ X-\hat{g}_T^{\mathcal {K}(N)}(A_T(Z)) \big | \mathcal {F}_t\right] \right) ^2\right] \\&\quad \le \mathbb {E}_{\tilde{\mathbb {P}}} \left[ \mathbb {E}_{\tilde{\mathbb {P}}} \left[ \left( X- \hat{g}_T^{\mathcal {K}(N)}(A_T(Z))\right) ^2\big | \mathcal {F}_t \right] \right] \\&\quad = \mathbb {E}_{\tilde{\mathbb {P}}} \left[ \left( X- \hat{g}_T^{\mathcal {K}(N)}(A_T(Z))\right) ^2\right] , \end{aligned}$$

where the first inequality follows from Jensen’s inequality and the last equality uses the projection law of expectations.

## Path-dependent and high-dimensional target functions

By now, we have discussed several aspects of portfolio replication and LSMC, which have highlighted some of the advantages of one method over the other. What we have not addressed so far is that the problems in portfolio replication and LSMC may differ very much in nature. In portfolio replication, the initial objective is to find the representation that best mirrors the payoff function. From that, the representation of the conditional expectation function is derived. In LSMC, only the conditional expectation function is approximated. Now, in many cases, the conditional expectation function differs in its structure, smoothness and dimensionality from the payoff function, where with smoothness we refer to the differentiability of the function. In particular, in life insurance, we may expect the payoff function to exhibit multiple kinks due to options and guarantees. Moreover, life insurance policies are often strongly path-dependent, which affects the dimensionality of the problem. The conditional expectation function typically “smoothes” the payoff function in terms of its differentiability, but also lowers its dimensionality compared to a path-dependent kinked payoff function. The difference in the structure, smoothness and dimensionality of the target function to be approximated significantly affects the feasibility of the LSMC and portfolio replication method in pratice. In this section, we highlight this point by means of several examples. We will see that the major challenge in portfolio replication compared to LSMC pertains to the replication of path-dependent payoff functions.

Finding either an LSMC representation or a replicating portfolio for a particular target function *X* with conditional expectation function $\mathbb {E}_{\tilde{\mathbb {P}}}[X | \mathcal {F}_t]$ requires two important steps before calibration.

1. Identification of all risk factors that drive the target function, summarized by $A_t(Z)$ and $A_T(Z)$, respectively.
2. Choosing a basis build on $A_t(Z)$ in LSMC and choosing a basis build on $A_T(Z)$ in portfolio replication.

We will elaborate on these two in the remainder of this section. The first step in LSMC and portfolio replication is the identification of all risk factors that drive the target function, for which a basis representation shall be found. In portfolio replication, this means that the risk factors of $g_T$ must be identified, while in LSMC, the risk factors driving $g_{0,t}$ must be determined. The complexity of finding the LSMC or replicating portfolio solution depends greatly on the number and type of risk drivers underlying the target function. Recall from Sect. 2 that the dimensionality of $A_t(Z)$ and $A_T(Z)$ is denoted by $\ell _t$ and $\ell _T$, respectively, which we view as an indicator for the complexity of the problem. The following examples illustrate the identification of $A_t(Z)$ and $A_T(Z)$, respectively (see also [7, 8]).

### Example 13

(Asian option)

Let *Z* be one-dimensional and consider a discrete Asian option on a stock with

$$\begin{aligned} X=\max \left( \frac{1}{T} \sum _{s=1}^T Z_1(s) - K, 0 \right) , \end{aligned}$$

where *K* is the strike price. The payoff *X* of the contingent claim depends on all past stock values prior to maturity. $A_T(Z)$ must now comprise all the information of the underlying driver such that *X* is specified. We may choose $A_T(Z)$ as the time average over the past stock values, which suffices to calculate the payoff *X*. Then, *X* only depends on $\sum _{s=1}^T Z_1(s)$. Thus, $A_T(f) = \left( \sum _{s=1}^T f(s) \right)$ for every function $f \in \mathbb {D}[0,T]$ and therefore $\ell _T =1$. Alternatively, we may also choose to take into account the value of $Z_1(s)$ at each time point, i.e. $A_T(f) = \left( f(1),\ldots ,f(T)\right)$ leading to $\ell _T=T$.

The conditional expectation function at time $t < T$, $\mathbb {E}_{\mathbb {Q}}\left[ X(T)|\mathcal {F}_t \right]$, only depends on $\sum _{s=1}^t Z_1(s)$ and $Z_1(t)$. Hence, $\ell _t=2$.

### Example 14

(European Basket Option)

Consider a *d*-dimensional basket option of the type

$$\begin{aligned} X=\max \left( \sum _{i=1}^d Z_i(T)-K, 0 \right) , \end{aligned}$$

where *K* is the strike price. In order to identify *X*, it suffices to take $A_T(f)=\sum _{i=1}^d f_i(T)$ for every function $f \in \mathbb {D}[0,T]$ and therefore $\ell _T=1$. Alternatively, we could also take $A_T(f) = f(t)$ for every function $f \in \mathbb {D}[0,T]$ leading to $\ell _T=d$.

Now, let us take a look at the conditional expectation function. In general, $\mathbb {E}_{\tilde{\mathbb {P}}}\left[ X(T)|\mathcal {F}_t \right]$ depends on $\mathbf {Z}(t)=(Z_1(t),\ldots ,Z_d(t))$ and not only on $\sum _{i=1}^d Z_i(t)$. Then, $A_t(f)=f(t)$ for every function $f \in \mathbb {D}[0,t]$ and therefore $\ell _t=d$. We give an example that shows our claim. Consider two assets $Z_1(t)$ and $Z_2(t)$ that move across the time steps $t=0,1,2$ as outlined in the trees below.
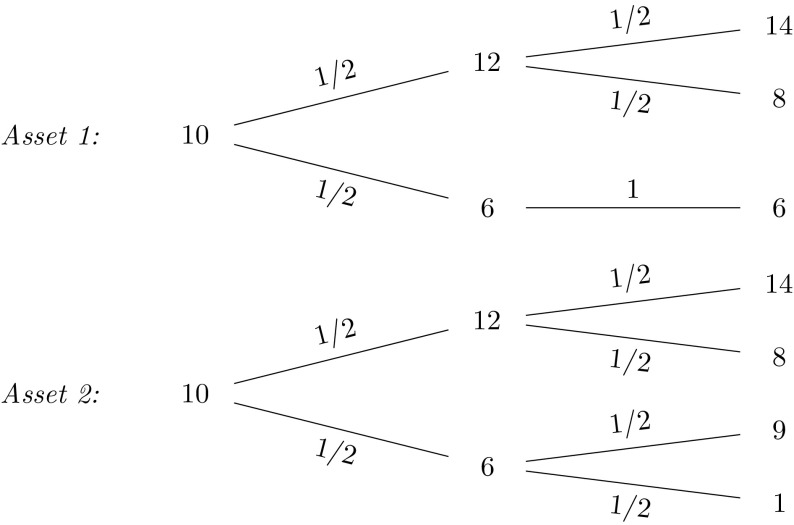


Take $X= \left( Z_1(2)+Z_2(2)- K \right) ^{+}$ with $K=10$. We are interested in the conditional expectation at time $t=1$, i.e. $\mathbb {E}_{\mathbb {P}}[X | \mathcal {F}_1]$, for which we obtain the following results

$$\begin{aligned}&\mathbb {E}_{\mathbb {P}}[X |Z_1(1)= 12, Z_2(1)=12 ] = 12 \\&\mathbb {E}_{\mathbb {P}}[X |Z_1(1)= 12, Z_2(1)= 6 ] = 6.25\\&\mathbb {E}_{\mathbb {P}}[X |Z_1(1)= 6, Z_2(1)=12 ] = 7\\&\mathbb {E}_{\mathbb {P}}[X |Z_1(1)=6 , Z_2(1)= 6 ] = 2.5. \end{aligned}$$

We immediately see that knowing the sum $Z_1(1)+Z_2(1)$ at time $t=1$ does not suffice to uniquely determine the conditional expectation at time $t=1$. In particular, for $Z_1(1)+Z_2(1) = 18$ the conditional expectation can either be 6.25 or 7.

### Example 15

(*Profit-sharing contract*)

Consider a profit-sharing contract, in which on a yearly basis interest is credited to the policyholder’s account. A minimum crediting rate is guaranteed and additional profit is shared depending on the specification of the bonus credited. Then, the contingent claim *X* at maturity *T* is given by

4.1 $$\begin{aligned} X=L_0 \prod _{s=1}^T (1+r_G+r_B(s)), \end{aligned}$$

where $L_0$ is the initial value of the policy, $r_G$ denotes the minimum guarantee rate and $r_B(s)$ is the bonus credited at time *s*. Let $r_B(s) = (r_A(s)-r_G)^{+}$, where $r_A(s)$ denotes the time *s* return of some reference portfolio. Straightforwardly, we can define $A_T(f) = \prod _{s=1}^T (1+r_G+f(s))$ for every function $f \in \mathbb {D}[0,T]$ and therefore $\ell _T=1$. As a result, the dimensionality of the problem is only one, but the specification of $A_T(Z)$ is complex. In that respect, we can also specify $A_T(f) = \left( f(1), \ldots , f(T)\right)$ and therefore $\ell _T=T$.

The conditional expectation function $\mathbb {E}_{\tilde{\mathbb {P}}}[X | \mathcal {F}_t]$ in general depends on

$$\left( \prod _{s=1}^t (1+r_G+f(s)) \right) $$ and $$r_B(t)$$. Therefore, $$\ell _t = 2$$.

The previous examples have shown that for the same problem statement the complexity of the LSMC and portfolio replication method in terms of the dimensionality of the problem may be quite different. Moreover, for the replicating portfolio technique, we have stressed that in principle different $A_T(Z)$ can be constructed.^4^ For a path-dependent insurance policy, we can either choose the state vector such that it captures the path-dependency or include each element on the path. There is, however, a major trade-off in choosing a lower-dimensional $A_T(Z)$ over a higher-dimensional $A_T(Z)$, which we want to point out next.

Given $A_t(Z)$ and $A_T(Z)$ are identified, a suitable basis must be chosen, which is constructed on the underlying risk factors, i.e. on $A_t(Z)$ and $A_T(Z)$, respectively. In the replicating portfolio problem, we moreover require basis functions, for which the conditional expectation under the relevant measure can be fairly easily and quickly determined, preferably even closed-form. For the pure replication of the payoff function *X*, the path-dependency of *X* may well be captured by either choice of $A_T(Z)$. However, once the conditional expectation of *X* is obtained by applying the conditional expectation operator to the basis on the path-dependent $A_T(Z)$, the original dilemma of valuing *X* at time *t* is transferred to the problem of valuing the basis. Hence, the more complex the underlying risk factor $A_T(Z)$ the more difficult it will be to obtain a closed-form solution to the conditional expectation of that basis. Choosing a lower-dimensional but more complex $A_T(Z)$ may therefore complicate the ease of determining the time *t* value of the basis build on it. To that end, using vanilla-style basis functions, i.e. functions on path-independent risk drivers, to replicate path-dependent insurance claims has the disadvantage of producing a high-dimensional $A_T(Z)$, but the advantage that the conditional expectation of the basis is easily available.

The LSMC method offers two advantages here over the portfolio replication method. First, its basis must not be valued under the conditional expectation operator, meaning that a complex structure for $A_t(Z)$ triggers no successive difficulties. Therefore, a low-dimensional $A_t(Z)$ with complex (path-dependent) structure can always be chosen. Second, as Example 15 has shown, $A_t(Z)$ is potentially lower-dimensional than $A_T(Z)$ if a composite (but low-dimensional) $A_T(Z)$ results in a too complex valuation of the basis build on it. As the discussion highlights, finding a basis is a much easier task in LSMC than in portfolio replication.

Let us now consider the construction of a multivariate basis and show why the dimensionality $\ell _t$ and $\ell _T$, respectively, matter. The linear sieve approximation to multivariate contingent claims is obtained analogously to the univariate representation by constructing a tensor product space, as described in [14]. Accordingly, the multivariate orthonormal basis is constructed by the tensor product of the respective univariate basis. While the basis is still countable, it is much more elaborate. Truncating the basis representation at *K* in the univariate case would give $K^{\ell }$ basis terms in the multivariate case of dimension $\ell$. Thus, the higher-dimensional $A_t(Z)$ in LSMC and $A_T(Z)$ in portfolio replication, the larger the basis. The curse of the dimensionality problem quickly dominates. Consider the profit-sharing policy contract of Example 15. For a life insurance policy, the terminal time point typically lies far in the future, say 30–60 years from now. Consider $T=30$ and $K=5$ basis terms per dimension. For portfolio replication $A_T(Z)$ is 30-dimensional resulting in $K^{\ell _T} = 5^{30} \approx 9.31 \times 10^{20}$ basis terms. In order to estimate such a tremendous number of coefficients, an immense sample size is required. Hence, in terms of the simulation effort, the problem becomes infeasible. Compare that to LSMC where $\ell _t=2$ leading with $K=5$ per dimension to $K^{\ell _t}=5^2=25$ basis terms.

When it comes to path-dependent target functions, the lower-dimensionality of the conditional expectation function and the indifference for the basis to be easily valued under the conditional expectation operator seems to give LSMC a competitive edge over portfolio replication. On the other hand, the curse of dimensionality in portfolio replication hits in when $A_T(Z)$ is chosen such that each of its components is path-independent and the basis is build as the tensor product of the univariate basis. Therefore, solutions for portfolio replication may be found by deviating from the strict framework of building a basis. In the next example, we discuss the construction of a replicating portfolio for a common path-dependent insurance contract.

### Example 16

(Grosen–Jorgensen profit-sharing contract)

In Example 15, a general profit-sharing contract is discussed. A well-known variant of profit-sharing contracts is the insurance contingent claim modeled in [22]. Here, the bonus return depends on the performance of the insurer’s underlying asset portfolio. The contract’s payoff at time *T* is as in (4.1) with the yearly bonus rate $r_B(s)$ defined as

$$\begin{aligned} r_B(s) = \max (0, \delta \left( \frac{A(s-1)}{L(s-1)} - (1+\lambda ) \right) -r_G ), \end{aligned}$$

where *A*(*s*) defines the underlying asset portfolio value and *L*(*s*) gives the value of the liabilities at time *s*. Note that, from Eq. (4.1), the liability value at time *s* is recursively calculated as

$$\begin{aligned} L(s) = L(s-1) (1+ r_G + r_B(s)). \end{aligned}$$

Then, $\lambda$ defines a buffer ratio and $\delta$ the fraction of the excess return that is shared with the policyholder. Clearly, the value of the liabilities at maturity depends on the performance of the underlying asset portfolio over time. Let us consider path-dependent basis functions on the yearly return of the asset portfolio, which captures much of the path-dependent dynamics of the Grosen–Jorgensen payoff, but is still different in its structure.

Consider a sequence of generalized Asian options on the asset process *A*(*s*) as basis, where the $k^{\text {th}}$ basis is defined as follows.

4.2 $$\begin{aligned} e_k(\varvec{A}) = \max (0,\varvec{a}_k^T \varvec{A} - d_k), \; k=1,...,K, \end{aligned}$$

where $\varvec{A} = (A(1),\ldots ,A(T-1))^T$ refers to the underlying asset process over time,

$\varvec{a}_k = (a_{k,1},\ldots ,a_{k,T-1})^T$ is a series of coefficients for the calculation of the weighted average and $d_k$ is the strike. Moreover, for the first basis term, we take

$$\begin{aligned} e_0 (\varvec{A}) = \varvec{a}_0^T \varvec{A} \end{aligned}$$

with $\varvec{a}_k = (a_{0,1},\ldots ,a_{0,T-1})^T$ a $(T-1)$-vector of coefficients. The parameters $\lbrace {\varvec{a}_{k}}, {d_{k}} \rbrace$, $k=0,\ldots ,K$ are determined by minimizing the sum of squared errors. The replicating portfolio $R_{P}$ is then given by

4.3 $$\begin{aligned} R_{P}({\varvec{A}}):= {\sum _{k=0}^K} {e_{k}}(\varvec{A}). \end{aligned}$$

Clearly, the structure of the generalized Asian options does not fully identify the original Grosen–Jorgensen payoff. Nonetheless, as we will see, with the replicating portfolio of (4.3) the behaviour of the Grosen–Jorgensen payoff can be largely captured.

To empirically test the performance of generalized Asian options, we consider a Grosen–Jorgensen payoff with maturity $T=11$, $r_G=0$, $L_0=A_0=100$, $\lambda =0.1$ and $\delta =0.75$. Let the asset process be given by a geometric Brownian motion

$$\begin{aligned} A(s) = A(s-1) e^{(\mu -\frac{1}{2} \sigma ^2) + \sigma (W(s) - W(s-1) )} \end{aligned}$$

**Fig. 30:**
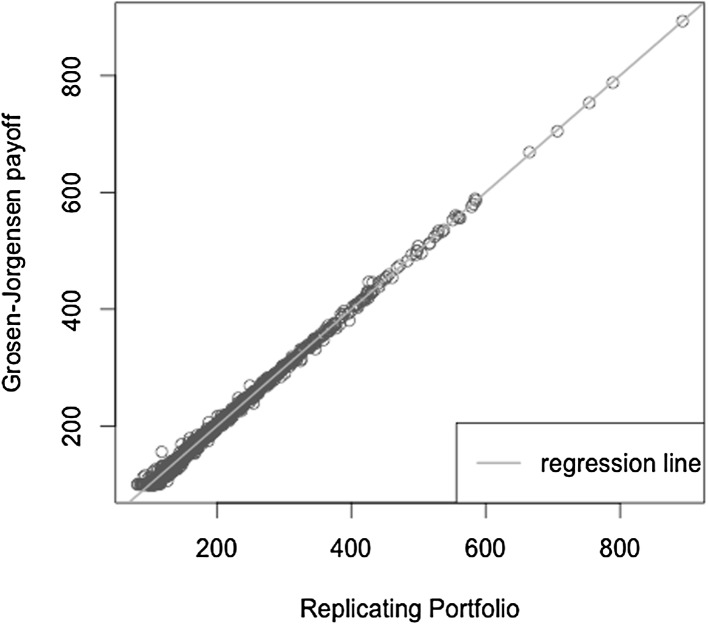
Goodness of fit of the replicating portfolio (Example 16)

**Fig. 31:**
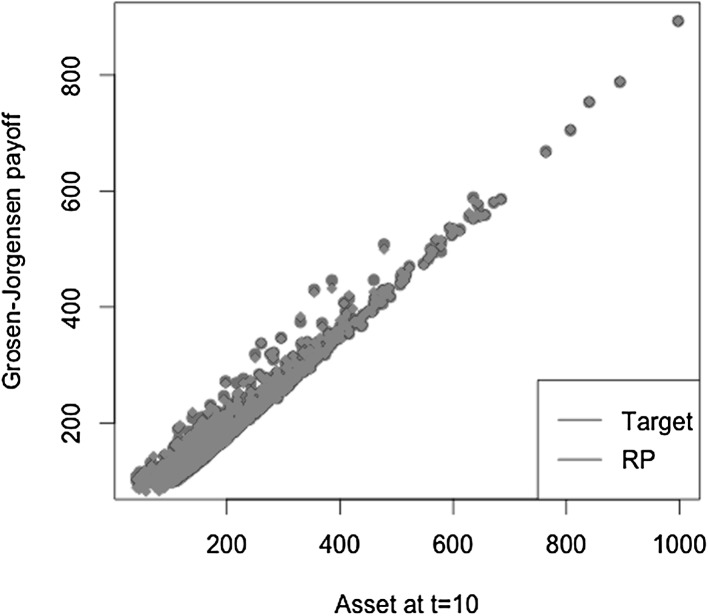
Comparison of variation with asset process at $t=10$ (Example 16)

**Fig. 32:**
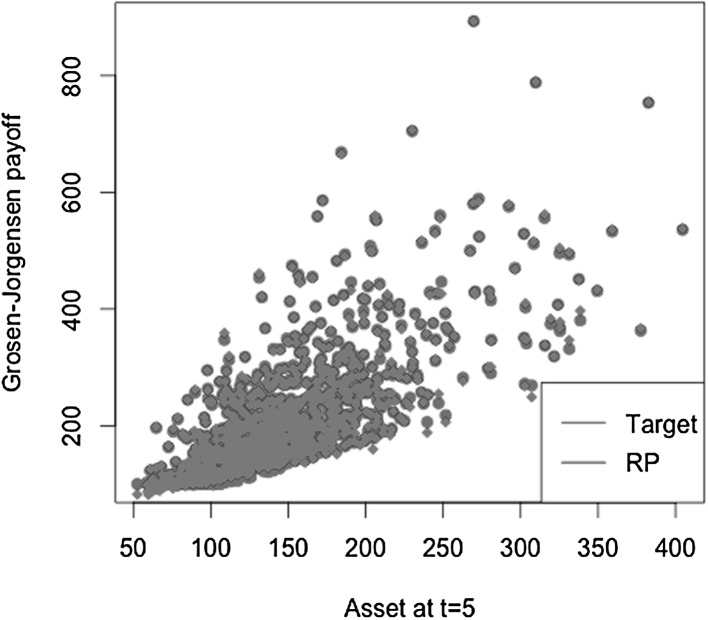
Comparison of variation with asset process at $t=5$ (Example 16)

**Fig. 33:**
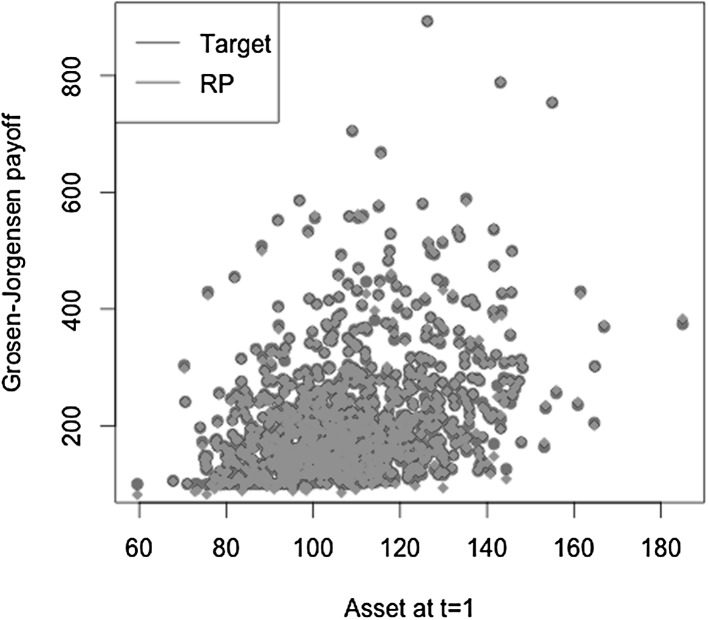
Comparison of variation with asset process at $t=1$ (Example 16)

In our example $\mu =0.08$ and $\sigma =0.16$. Based on a sample of size $N=1000$ the coefficients of the replicating portfolio in (4.3) are globally optimized for a chosen number of basis terms *K* by minimizing the error sum of squares. For $K=4$ , a remarkably good fit is already achieved with an out-of-sample $R^2$ of $99.73\, \%$, which is illustrated in Fig. 30. The out-of-sample $R^2$ is based on a sample $m=1000$ that has not been used in the calibration of the replicating portfolio. Figures 31, 32 and 33 illustrate the variation of the Grosen–Jorgensen payoff function and the replicating portfolio against the asset process at different time points. Clearly, the replicating portfolio mirrors the behavior of the target payoff very closely.

Recall that, in the context of insurance risk capital calculations, replicating portfolios of the liability payoffs are constructed in order to simplify the calculation of the liability value at the risk horizon. Therefore, the value of the replicating instruments making up the replicating portfolio must be readily available. Generalized Asian options are path-dependent and closed-form solutions to their value are normally not available. However, good approximations to the value of Asian options have been found, which makes them almost analytically priceable and justifies their use in portfolio replication. We refer the reader to, for example, [37].

The previous example has shown that, although portfolio replication is a more difficult problem when it comes to path-dependent insurance products, good solutions can be found and portfolio replication is feasible for such payoff functions.

## Conclusion

**Table 5:** Comparison portfolio replication versus LSMC

Portfolio replication (LSMC regress-later)	LSMC (LSMC regress-now)
Non-noisy regression	Noisy regression
By construction, implies fit to the conditional expectation function at any $t < T$	Only achieves a fit to the conditional expectation function $\mathbb {E}_{\tilde{\mathbb {P}}}[X | \mathcal {F}_t]$ for a particular $t < T$
$R^2$ is a useful measure with an $R^2=1$ reflecting a perfect fit	$R^2$ is not a useful measure and is always lower than 1
Result is asymptotically independent of the measure used for calibration	Result depends on the measure chosen for calibration
Potentially faster convergence rate than $N^{-1}$ can be achieved	Convergence rate can never exceed $N^{-1}$
Path-dependent policies imply a higher dimensionality of the problem and finding a good basis is more challenging	Path-dependent policies do not imply a higher dimensionality of the problem in LSMC. Finding a basis is in principle easier
Choice of basis is critical. The conditional expectation of the basis must be readily available	Choice of basis is not limited by the easiness of calculating its conditional expectation. In principle, any basis build on $A_t(Z)$ works

In this paper, two popular proxy techniques commonly applied in the risk management of life insurance policies for approximating unknown conditional expectation functions have been discussed. Their mathematical set-ups have been given and it has been shown that, while both methods belong to the category of least squares Monte Carlo algorithms, they work very differently. LSMC provides a direct approximation to the conditional expectation function and is a function-fitting method. In portfolio replication, a replicate of the terminal payoff function is constructed instead. This is then used to obtain a proxy to the conditional expectation function. The difference in the set-up of LSMC and portfolio replication has multiple practical consequences which have been illuminated using elementary examples. In that respect, it has been shown that the performance of LSMC versus portfolio replication depends on several factors. These are summarized in Table 5. Clearly, portfolio replication enjoys multiple benefits such as potentially faster convergence than at rate $N^{-1}$, where *N* is the sample size, asymptotic measure independence and $R^2$ as a simple and meaningful measure for assessing the quality of the replicating portfolio. Its major challenge pertains to the replication of (strongly) path-dependent insurance policies. Using a “naive” multivariate basis constructed as the tensor product of the univariate bases quickly poses the curse of a dimensionality problem. The LSMC technique does not suffer from the same poblem and is easier to use for path-dependent payoffs compared to the replicating portfolio technique. In portfolio replication, alternative basis constructions must be considered in order to overcome the curse of a dimensionality problem. For a strongly path-dependent profit-sharing contract commonly encountered in insurance, we have provided a solution approach that results in a very good replicating portfolio. Of course, much room for future research is left to explore the possibilities for replicating path-dependent insurance payoffs. Overall, we can conclude that, while portfolio replication is a more difficult problem when it comes to path-dependent payoff functions, we have revealed multiple advantages of portfolio replication which show that the challenge of finding a replicating portfolio is worthwhile.

## Footnotes

1 We also remark that in the case where the basis includes a constant $\mathbb {E}_{\tilde{\mathbb {P}}}[a_{0,t}^K(A_t(Z))]=0$.

2 We remark again that in the case where the basis includes a constant $\mathbb {E}_{\tilde{\mathbb {P}}}[a_T^K(A_T(Z))]=0$.

3 This means that *K* should be increased, i.e. more basis terms build on the already identified risk factor. We remark that a higher *K* in principle also requires a larger sample size as more parameters need to be estimated. The relationship of *K* and *N* is also addressed in Sect. 3.5 in the context of the asymptotic convergence properties of LSMC and portfolio replication.

4
${^{4}}$While theoretically the same holds for $A_t(Z)$, we omit this as for $A_t(Z)$ we may always choose the lowest dimensionality since we do not face the pricing dilemma of the basis.
